# An atlas of genome-wide gene expression and metabolite associations and possible mediation effects towards body mass index

**DOI:** 10.1007/s00109-023-02362-z

**Published:** 2023-09-06

**Authors:** Carl Beuchel, Julia Dittrich, Susen Becker, Holger Kirsten, Anke Tönjes, Peter Kovacs, Michael Stumvoll, Markus Loeffler, Andrej Teren, Joachim Thiery, Berend Isermann, Uta Ceglarek, Markus Scholz

**Affiliations:** 1 Institute for Medical Informatics, Statistics and Epidemiology, Leipzig University, Leipzig, Germany; 2 Institute of Laboratory Medicine, Clinical Chemistry and Molecular Diagnostics, Leipzig University, Leipzig, Germany; 3 Department of Forensic Toxicology, Institute of Legal Medicine, University Leipzig, Leipzig, Germany; 4 LIFE – Leipzig Research Center for Civilization Diseases, Leipzig University, Leipzig, Germany; 5 Medical Department III – Endocrinology, Nephrology, Rheumatology, University Hospital Leipzig, Leipzig, Germany; 6 Deutsches Zentrum für Diabetesforschung, Neuherberg, Germany; 7 Heart Center Leipzig, Leipzig, Germany

**Keywords:** Acylcarnitine, Amino acid, Gene expression, Mediation analysis

## Abstract

**Abstract:**

Investigating the cross talk of different omics layers is crucial to understand molecular pathomechanisms of metabolic diseases like obesity. Here, we present a large-scale association meta-analysis of genome-wide whole blood and peripheral blood mononuclear cell (PBMC) gene expressions profiled with Illumina HT12v4 microarrays and metabolite measurements from dried blood spots (DBS) characterized by targeted liquid chromatography tandem mass spectrometry (LC–MS/MS) in three large German cohort studies with up to 7706 samples. We found 37,295 associations comprising 72 amino acids (AA) and acylcarnitine (AC) metabolites (including ratios) and 8579 transcripts. We applied this catalogue of associations to investigate the impact of associating transcript-metabolite pairs on body mass index (BMI) as an example metabolic trait. This is achieved by conducting a comprehensive mediation analysis considering metabolites as mediators of gene expression effects and vice versa. We discovered large mediation networks comprising 27,023 potential mediation effects within 20,507 transcript-metabolite pairs. Resulting networks of highly connected (hub) transcripts and metabolites were leveraged to gain mechanistic insights into metabolic signaling pathways. In conclusion, here, we present the largest available multi-omics integration of genome-wide transcriptome data and metabolite data of amino acid and fatty acid metabolism and further leverage these findings to characterize potential mediation effects towards BMI proposing candidate mechanisms of obesity and related metabolic diseases.

**Key messages:**

- Thousands of associations of 72 amino acid and acylcarnitine metabolites and 8579 genes expand the knowledge of metabolome-transcriptome associations.
- A mediation analysis of effects on body mass index revealed large mediation networks of thousands of obesity-related gene-metabolite pairs.
- Highly connected, potentially mediating hub genes and metabolites enabled insight into obesity and related metabolic disease pathomechanisms.

**Supplementary Information:**

The online version contains supplementary material available at 10.1007/s00109-023-02362-z.

## Introduction

Advances in high-throughput metabolomics make the blood metabolome a readily available and cheap target to characterize large studies and cohorts and to conduct large-scale multi-omics investigations [1]. Obesity is a highly heterogeneous metabolic disease that increases risk for other non-communicable diseases [2]. The link between obesity and a wide range of metabolic disorders such as insulin resistance and type 2 diabetes mellitus (T2D) has long been established [3].

Metabolic alterations have been studied as causes and symptoms in these and other pathologies [4–6]. For example, the influence of branched-chain amino acids (BCAAs) on metabolic disease has been the subject of numerous studies [7–9]. Perturbations of acylcarnitine (AC) metabolism have also been studied in connection to insulin sensitivity and T2D and other metabolic disorders [10–13]. These studies provide a strong rationale of investigating the blood metabolome in relation to metabolic traits and diseases.

In the search for the genetic basis of metabolic traits, a large catalogue of genetic association studies identifying common and rare genetic variants influencing blood metabolites is available and is continuously extended [14–18]. Despite high heritability of metabolic traits, additional sources of variation, e.g., via transcriptional regulation, are of interest to create a more comprehensive picture of the variation of blood metabolites in humans. Additionally, instances of transcriptional regulation of metabolites as well as metabolic regulation of gene expression were described in relation to several diseases, such as a hypertriglyceridemia inducing effect of BCAAs via expression of a transcription factor [19, 20]. Although studies on the association of human blood metabolome and transcriptome are available [20–23], sample sizes are considerably lower as compared to genetic association studies. Moreover, heterogeneity of metabolomics platforms, such as NMR and liquid chromatography tandem mass spectrometry (LC–MS/MS), reduces comparability of results between studies. In this study, we perform the first and largest genome-wide gene expression/metabolite association meta-analysis of three independent studies, all utilizing the same metabolomics and microarray platforms. As an application of the resulting comprehensive association catalogue, we analyze possible mediation effects between gene expressions, metabolites, and BMI as an example metabolic trait to identify new candidates of causal mechanisms and relationships in obesity.

## Material and methods

### Study characteristics and design

We performed a genome-wide gene expression–metabolite association meta-analysis of the following three studies.

#### LIFE-Adult

For the population-based LIFE-Adult study, 10,000 age- and sex-stratified randomly selected individuals were recruited from the city of Leipzig, Germany [24, 25]. Phenotyping focused on civilization diseases and related risk factors. Samples were collected after an overnight fasting period. Paired gene expression (*n* = 3173) and metabolite (*n* = 9646) data were available for 3145 participants.

#### LIFE-Heart

Patients with suspected or confirmed coronary artery disease were collected at the Heart Center Leipzig, Germany [26]. All participants underwent coronary angiography. Confirmed coronary artery disease comprised stable disease as well as acute (AMI) or historical myocardial infarction. Paired gene expression (*n* = 4143) and metabolite (*n* = 5860) parameters were available for 3626 patients (including 1271 patients with AMI). A fasting period was not required prior to blood sampling. There was no sample overlap between the LIFE-Adult and LIFE-Heart studies.

#### Sorbs

Participants of the Sorb study were recruited from the self-contained Sorb population, an ethnic minority of Slavic origin from Upper Lusatia in Eastern Saxony, Germany [27, 28]. Blood samples were collected after an overnight fasting period of at least 8 h. Paired gene expression (*n* = 988) and metabolite data (*n* = 935) were available for 935 participants.

All studies comply with the ethical standards of the Declaration of Helsinki and with approval by the ethics committee of the University of Leipzig (LIFE-Adult: Reg. No 263-2009-14122009, LIFE-Heart: Reg. No 276e2005, Sorbs: Reg. No: 088-2005). LIFE-Heart was registered at ClinicalTrials.gov (No NCT00497887). All participants gave their written and informed consent.

Please see Supplemental Table S1 for the respective descriptive statistics of study participants.

### Metabolite measurement and pre-processing

#### Sample preparation

In the LIFE studies, 40 µl of EDTA whole blood was directly spotted on filter paper (WS 903 Schleicher and Schüll, Germany), dried for 3 h, and stored and − 80 °C till analysis. In the Sorb study, EDTA whole blood was not available. Therefore, 40 µl of the cell residue after plasma centrifugation was spotted on filter paper WS 903.

#### Mass spectrometric analysis

Punched-out 3-mm diameter blood spots (corresponding to 3 µl of blood) were extracted using methanol containing isotope-labeled internal standards and butylated in 96 well plates, as is described elsewhere [29–31]. Each plate contained two quality control samples for the estimation of the inter-assay coefficient of variation. This method was validated for the identification of relative concentration differences between different studies [31]. An API 2000 or API 4000 tandem mass spectrometer (SCIEX, Darmstadt, Germany) was applied for flow injection analysis. Quantitative metabolite variable data of 62 metabolites (27 amino acids, 34 acylcarnitines, and free carnitine) were derived using ChemoView 1.4.2 (Applied Biosystems, Germany). Additionally, we calculated a biologically relevant sum (total ACs) and 34 ratios of metabolites for assessment of reaction equilibria within physiological pathways involving these metabolites. Consequently, 97 metabolite features were available for analysis. See Supplemental Table S2 for an overview and the formulas of the derived ratios.

#### Metabolite pre-processing

Metabolite pre-processing was performed study wise. The workflow was developed and described elsewhere [32]. There, we demonstrated that applying this workflow minimized between-study heterogeneity caused, e.g., by different study designs or sampling techniques. Briefly, we removed metabolite measurements above of mean + 5 × SD of log-transformed data to account for skewness of data. Metabolite measurements at zero were temporarily excluded for this step only. A maximum of 0.3% of measurements were removed per metabolite and cohort by these criteria. Then, metabolites were inverse normally transformed. We batch-adjusted transformed quantities using an empirical Bayes method implemented in the R-function “ComBat” of the “sva” package [33, 34].

#### Relationship adjustment in Sorb cohort

We adjusted for relatedness among Sorb subjects by fitting a generalized linear model as implemented in the “polygenic()” R-function of the “GenABEL” package [35]. The required kinship matrix was estimated using available SNP microarray data [27, 36].

### Gene expression measurement and pre-processing

#### Sample collection, pre-analytics, and measurement

In LIFE-Heart, RNA of 4143 patients was extracted from peripheral blood mononuclear cells (PBMCs). Details of RNA extraction can be found elsewhere [37, 38]. In the Sorb study, 988 samples of PBMCs were isolated from blood samples. The full sample preparation procedure is described elsewhere [39]. For LIFE-Adult participants, 3173 whole blood samples were collected and stored at − 80 °C in Tempus Blood RNA Tubes (Life Technologies). See the Supplemental Methods for a detailed description of the sampling and measurement process in each study.

RNA measurement was performed using Illumina HT-12 v4 Expression BeadChips (Illumina, San Diego, CA, USA) and scanned on the Illumina iScan according to the manufacturer’s specifications [37].

#### Data pre-processing

We pre-processed the whole blood and PBMC gene expression profiles of the three studies separately, applying the workflow implemented in the “HT12ProcessoR” R package (https://github.com/holgerman/HT12ProcessoR) that uses Bioconductor functionality [40]. The workflow is described in detail in Kirsten et al. and in the Supplemental Methods [41]. In brief, the data are log_2_ transformed and quantile normalized. We adjusted for technical batch effects (sentrix barcode) by applying “ComBat” [33, 34]. We removed expression probes not expressed in more than 5% of samples as well as probes significantly associating with batches after Bonferroni correction. We also removed samples with low quality based on the number of expressed genes. Next, we applied a filter based on the Mahalanobis distance of specific Illumina probes designed for quality analyses. Lastly, we removed subjects with a Euclidean distance of expression values of larger than four times the range between the 25th and 75th percentiles (interquartile range (IQR)) from the median. We mapped probes to unique genes via the “Ingenuity Pathway Analysis” database (QIAGEN Inc.). Probes that could not be mapped to a gene were removed. Sample and probe exclusions due to quality reasons are detailed in the Supplemental Methods and Supplemental Table S9. We adjusted for relatedness among Sorb subjects using the same method as for the metabolite data (see the “Metabolite measurement and pre-processing” section).

### Analysis of cofactors

In a previous study, we investigated the effects of 29 clinical and lifestyle factors on whole blood metabolites in our studies (Supplemental Table S1) [32]. It revealed that the following covariates have a relevant impact: age, BMI, sex, hours fasted (not available in the Sorbs), type 2 diabetes mellitus (T2D), hematocrit, and neutrophil percentage. Regarding blood gene expression, most important cofactors comprise age, sex, and the percentages of neutrophils and monocytes (Supplemental Fig. S1) [41]. We here considered the union of identified strong cofactors of metabolites and gene expression as potential confounders which is liberal in the sense that relevant confounders must affect both omics layers. We refrained from adjusting for BMI in the association analysis in order to preserve effects on metabolites and gene expression for the subsequent mediation analysis. For the same reason, we also did not adjust for diabetes status due to its high correlation with BMI. Thus, six factors were considered as covariates in the regression analysis. Since metabolite and gene expression data of AMI patients of LIFE-Heart are clearly affected by the acute situation, we decided to analyze these patients separately, i.e., two analysis groups were defined in LIFE-Heart, namely AMI and non-AMI.

### Single study association analysis

All analyses were performed on the subset of study subjects with complete gene expression, metabolite, and covariable information. We calculated associations of gene expression with metabolite levels for each study separately using multiple linear regression with the gene expression as dependent and the metabolite and covariates as independent variables. We used the functions “lmFit()” and “eBayes()” of the “limma” R package to carry out the association analysis in all studies [42].

As recommended [43, 44], multiple testing correction is performed hierarchically, i.e., we corrected *p*-values for multiple testing within each metabolite first (local adjustment). In a second step, smallest adjusted *p*-values of each metabolite are adjusted across metabolites (global adjustment). We used the Benjamini–Hochberg correction [45] for both local and global adjustment to control the false discovery rate (FDR) at 5% (R-function “p.adjust()” with “method = ”BH””). Analysis steps are outlined in Supplemental Fig. S2.

### Meta-analysis

Single study association summary statistics from the four subgroups (LIFE-Adult, LIFE-Heart non-AMI, LIFE-Heart-AMI, Sorbs) were meta-analyzed by a random effects model (REM) to account for the heterogeneity of sample processing and tissues used for gene expression analysis [46]. Study heterogeneity was assessed by I^2^ metrics. Again, we applied hierarchical multiple testing correction to determine significance of metabolite to gene expression association meta-analysis results. Only association results available in more than one study were meta-analyzed, resulting in a total of 26,042 probes which could be mapped to 17,735 unique genes.

To assess the extent of gene expression/metabolite associations, we estimated the proportion of null *p*-values (*η*_0_) from the empirical distribution of *p*-values. The proportion of non-null *p*-values was calculated as *η*_1_ = 1 − *η*_0_. We estimated *η*_0_ using “fdrtool” function “pval.estimate.eta0()” with the argument “method = ”smoother”” for each metabolite using REM *p*-values [47, 48].

Sample sizes of studies (see Table 1) allow detection of explained variances of metabolite/gene expression relationships of 4.4%, 3.2%, 1.7%, 1.2%, and 0.5% within the Sorb study, LIFE-Heart (AMI), LIFE-Heart (non-AMI), LIFE-Adult, and the overall meta-analysis, respectively, with a power of 80% and a significance cutoff of *α* = 2.0 × 10^−8^ accounting for a multiple testing correction of approximately 2.5 million tested metabolite/gene expression pairs (PASS 2020).

**Table 1:** Overview of total association results per study and in the subsequent meta-analysis

	**LIFE-Adult**	**LIFE-Heart (AMI)**	**LIFE-Heart (non-AMI)**	**Sorbs**	**Meta-analysis**
**N**	3145	1271	2355	935	7706 (max.)
**Total number of tested associations**	2,072,696	2,275,329	2,315,778	2,250,303	2,526,074
**Total significant associations (FDR 5%)**	247,010	19,252	19,349	48	37,461
**Estimated proportions of true positive findings (*η***_**1**_**)**	0.3	0.14	0.11	0.03	0.076
**Associating probes**	18,378	6174	5716	31	10,517
**Associating transcripts**	13,674	5204	4898	28	8579
**Associating metabolites**	67	53	74	10	72

The number of associating gene expression probes, respectively, mapped genes as well as metabolites is listed for each study and the meta-analysis as well. Additionally, the percentage of true positive associations, estimated from the distribution of *p*-values, is provided

In the association meta-analysis, we jointly analyzed gene expression measurements from whole blood, as well as PBMCs. To assess comparability of gene expression source tissues (whole blood in LIFE-Adult on one hand and PBMCs in LIFE-Heart and the Sorb study on the other hand), we conducted a separate meta-analysis considering only PBMC gene expression data (LIFE-Heart AMI/non-AMI and the Sorb study). We compared heterogeneity in the analyses (whole blood and PBMCs vs. PBMCs only) by comparing respective I^2^ estimates and respective random effects meta-analysis estimates.

### Mediation analysis

We tested for mediation effects considering the association triangles of gene expression, metabolites, and BMI. At this, BMI was always considered as outcome, while gene expression and metabolites were considered as exposure and mediator, respectively, or vice versa (Supplemental Fig. S3). The effect estimate of the outcome-mediator association conditional on the exposure is called the indirect effect, whereas the effect of the outcome-exposure association conditional on the mediator is called the direct effect [49]. The sum of direct and indirect effects is considered the total effect. Conceptually, a mediation analysis is a so-called third-variable analysis, in which the effect of a variable (called mediation effect of the mediator) is analyzed in respect to the relationship of two other variables, i.e., the association between exposure and outcome. A third-variable effect can only be interpreted as a mediation effect if the underlying causal/temporal order is assumed to be correct [50]. Thus, identified relationships can only be considered as candidates for causal/mechanistic effects.

Required association statistics for calculating mediation effects (resulting from the regression analyses (1) BMI ~ metabolite + covariates, (2) BMI ~ gene expression + covariates, (3) metabolite ~ gene expression + covariates, (4) gene expression ~ metabolite + covariates, and (5) BMI ~ metabolite + gene expression + covariates) were determined by meta-analyses of the three studies (see the “Meta-analysis” section) as follows. Single study associations were calculated by linear models (R function “lm()” in the “stats” package). We adjusted for the relevant covariates as described above (see the “Analysis of cofactors” section). BMI was log transformed. Continuous covariates and all gene expression values were scaled to mean *µ* = 0 and standard deviation *σ* = 1. Metabolites were scaled as a result of the inverse normal transformation. Single study effects were again combined by random effects meta-analysis. We applied correction for multiple testing using the same hierarchical procedure with the predictor variable as the family variable and local as well as global adjustment using the Benjamini–Hochberg procedure [44].

Mediation analysis is restricted to all pairs of gene expression probes and metabolites that were significantly associated in the meta-analysis. Since interactions between mediator and exposure regarding BMI outcome can result in invalid results [51, 52], we searched for such pairs by linear model analyses: BMI ~ metabolite + gene expression + metabolite*gene expression + covariates. Interaction effects of single studies were again combined via meta-analysis. Pairs with significant interactions were removed (hierarchical FDR = 5%).

Next, exposure and outcome are required to be significantly associated (total effect, *γ*_1_) or a significant direct effect (*β*_1_) should be present (effect of exposure on outcome adjusted for the mediator) [50]. We like to remark that in the latter case, only partial mediation could be tested, i.e., whether the direct effect size of the exposure to the outcome is reduced when adjusting the outcome for the mediator. In the first scenario, also complete mediation could be tested, i.e., absence of a direct effect. Due to the complexity of human metabolic pathways, complete mediation scenarios are expected to be rare [53]. Thus, we focused on partial mediation in the present analysis.

The mediation effect is determined by the product of beta estimates of the effect of the exposure on the mediator (*α*) and the mediator on the outcome (*β*), i.e., *β*_mediation_ = *α* × *β* [54–56]. In more detail, the product of the two *z* statistics of the beta estimates is calculated and compared to the distribution of the product of two standard normal distributions representing the expected distribution under the null hypothesis and used to calculate a *p*-value of the mediation effect. The R-function “pprodnormal()” of the “RMediation” package [57] was used for that purpose. Confidence intervals of the mediation effects were computed using the function “medci()” with Monte Carlo method as recommended [58]. Correction for multiple testing was again performed in a hierarchical way considering the exposures as families for local adjustments.

Finally, the proportion of the total effect that was mediated is calculated, i.e., PM = *α* × *β*/*α* × *β* + *τ*’, which is the ratio of the indirect effect of the exposure on the outcome and the total effect (*β*_total_ = *α* × *β* + *τ*’, i.e., sum of indirect (αβ) and direct (*τ*’) effects [56, 59]). This quantity will be used to distinguish between mediation directions, i.e., the mediated proportion of the total effect (PM) will be compared for the two possible causal effect directions gene expression → metabolite (GE → M), respectively, metabolite → gene expression (M → GE), if both mediations are significant (Supplemental Fig. S3). In more detail, we classified mediations as “strongly mediated metabolite effects” (by gene expression effects) if the following conditions hold: (1) the metabolite effect on BMI was significantly mediated by the gene expression probe, (2) for the mediation effect it holds PM ≥ 0.2, and (3) for the reverse mediation it holds PM ≤ 0.2. Conversely, “strongly mediated gene expression effects” (by metabolite effects) are classified using the same conditions, but for mediations of gene expression effects by metabolite effects. With “strong bi-directional mediations,” we denote significant mediation effects and PM ≥ 0.2 for both directions. We classified mediations as “weak mediation” when they were significant but none of the above classifications are met.

To demonstrate utility and to increase confidence in our results, we explored mediated effects of known obesity-associated genes. For this purpose, we created a catalogue of 52 genes reported for association with BMI (Supplemental Table S3) [60, 61]. We screened our mediation results for these genes and selected mediations with a high mediated effect proportion (PM ≥ 0.2). To assess the functional plausibility of the mediations, we screened the literature for additional functional experiments considering respective transcript-metabolite pairs.

## Results

### Comparison of single study results

We calculated associations of 26,042 gene expression probes (17,735 annotated genes) and 97 metabolites (45 AAs, 44 ACs, and 8 mix quotients of ACs and AAs and the sum of ACs) in up to four data sets.

Due to the largest sample size, most significant associations were found in LIFE-Adult, while only a few associations were found in the Sorbs with the lowest sample size (Table 1).

A total of nine associations involving transcripts of four genes (*ABCG1*, *ALAS2*, *HBA1*/*HBA2*, and *HBB*) and five metabolites (total acylcarnitines, acetylcarnitine (C2), propionylcarnitine (C3), leucine/isoleucine, Q19:(Leu/Ile)/C3) were significant (hierarchical FDR = 5%) in all four studies, all with the same direction of effect. These were the associations of *ABCG1* with leucine/isoleucine, *HBA1*/*HBA2* with C2, *ALAS2* with C2, *HBA1*/*HBA2* with C3, *ALAS2* with C3, *HBB* with C3, *HBA1*/*HBA2* with total acylcarnitines, *ALAS2* with total acylcarnitines, and *ALAS2* with the ratio of leucine/isoleucine and C3 (see Supplemental Table S4 for an overview and Online Supplemental Table OS1 for the full summary statistics).

### 37,295 gene expression/metabolite associations found in meta-analysis

**Fig. 1:**
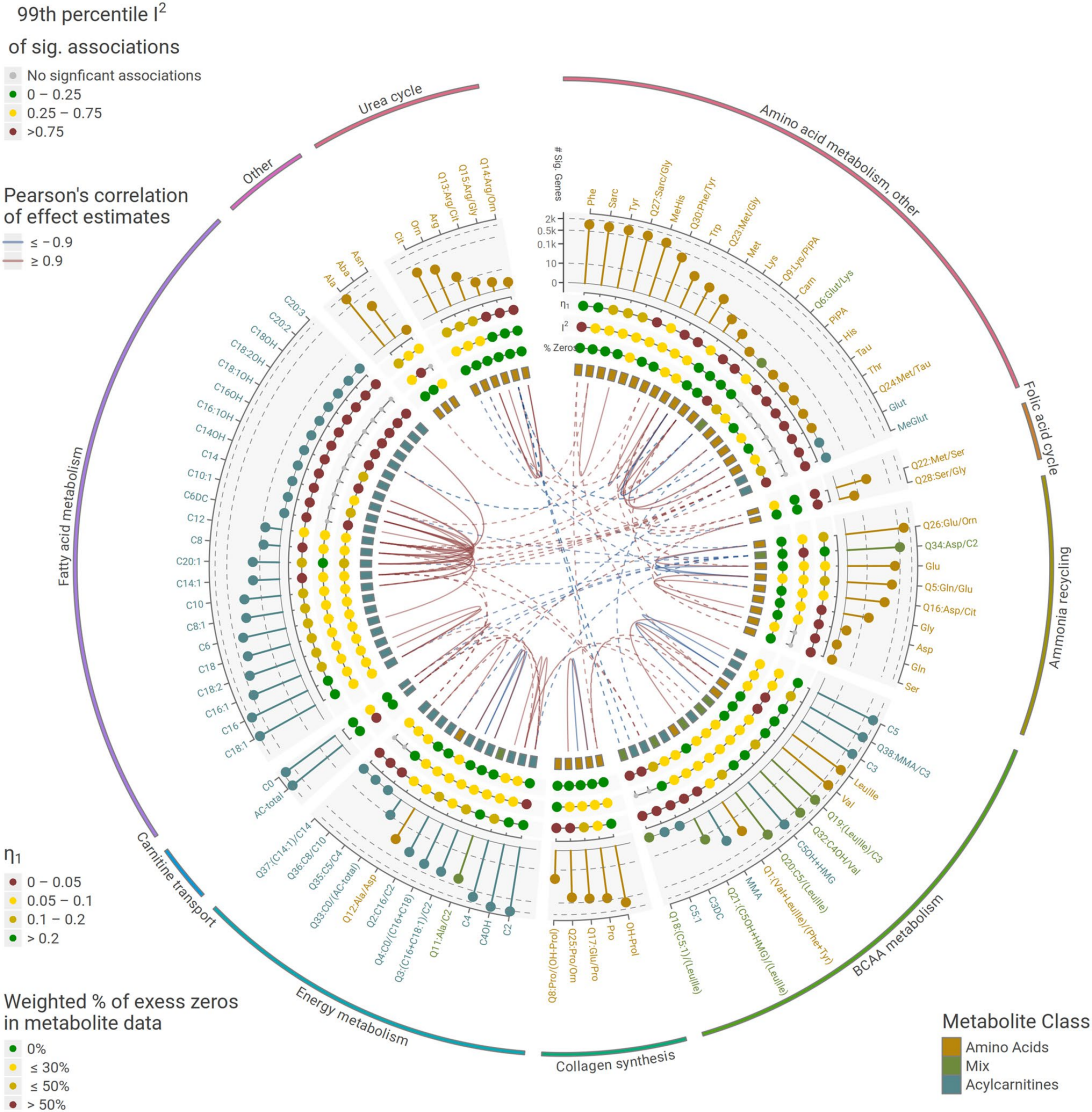
Overview of gene expression/metabolite associations found in meta-analysis. Twenty-six thousand forty-two gene expression probes (mapped to 17,735 unique genes) and 97 metabolites (45 amino acids, 44 acylcarnitines, and 8 mix quotients) were analyzed in our meta-analysis. Number of significant associations (at hierarchical FDR = 5%) are shown for each metabolite (middle ring), are grouped by their general metabolite pathway (outer ring), and colored according to class membership (amino acid, acylcarnitine and mix quotient, squares in the innermost ring). At the other inner dotted rings, the estimated fraction of true associations (*η*_1_), the 99th percentile of the estimated heterogeneity (I^2^) of associations across studies, and the percentage of zero values of metabolites are displayed as color-coded categories. Links between metabolites displayed in the center represent Pearson correlations (only correlations ≥ 0.9 or ≤  − 0.9 are shown) of the standardized effect estimates of the metabolite gene expression associations, thus indicating similar dependencies of metabolites on gene expressions. Links within metabolite pathways are represented by solid lines, links between pathway by dashed lines

An overview of metabolite-transcript associations significant in meta-analysis is shown in Fig. 1. Overall, 37,295 metabolite-transcript associations involving 72 (74.2%) metabolites and 8579 (48.4%) mapped genes were significant at hierarchical FDR = 5%. A total of 25 metabolites (8 AAs, 16 ACs, and 1 mix quotient) did not show any significant associations. Most of the metabolites without significant associations also showed a high percentage of (excess) zeros in the metabolite data, implying values below the detection limit and low abundance, and with it, low power to detect associations. Across-study heterogeneity of the associations as assessed by I^2^ statistics was moderate (Fig. 1). Only 262 (0.7%) of significant associations showed a relevant heterogeneity I^2^ ≥ 0.75.

The overall fraction of non-null hypothesis (*η*_1_) was estimated from the pooled amount of all tested hypothesis resulting in *η*_1_ = 0.076, i.e., we estimated that 7.6% gene expression/metabolite pairs are associated. When estimated separately for metabolites, *η*_1_ ranged from *η*_1_ = 0 for a total of 26 metabolites (5 AAs, 6 quotients, 15 ACs) to *η*_1_ = 0.313 for octadecenoylcarnitine (C18:1), i.e., this metabolite is likely regulated by many genes. Of all metabolites, C2 exhibited the largest number of associated transcripts (*n* = 2187). In terms of absolute standardized effect estimate ($\widehat{\beta }$), the eight strongest associating metabolites all belonged to the class of acylcarnitines. Alanine showed the strongest association among amino acids. Supplemental Table S2 shows the top transcripts for each metabolite, and the full summary statistics of the meta-analysis are available at Online Supplemental Table OS2.

On the gene expression level, most associations were observed for *BCL11A* (BAF chromatin remodeling complex subunit BCL11A), a C2H2 type zinc-finger protein, associating with 28 metabolites (13 AAs, 13 ACs, and 2 mix quotients; Supplemental Table S5). The gene with the strongest metabolite associations was *ALAS2* (5′-aminolevulinate synthase 2), whose associations with Sarc, C2, Q34, total ACs, and C3 exhibited the highest standardized effect estimates of all associations (Supplemental Fig. S4, Online Supplemental Table OS2).

**Fig. 2:**
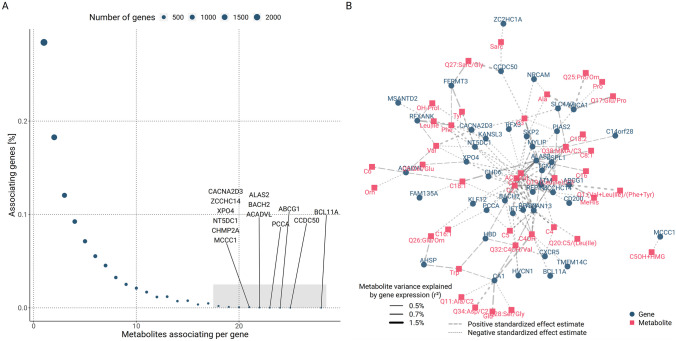
Detailed analysis of genes showing multiple associations. We display the degree of connectivity of transcript-metabolite associations, considering 46 hub genes associated with the highest number of metabolites (≥ 18) in our meta-analysis. **A** We present the frequency of genes with a prescribed number of significantly associating metabolites. The gray area corresponds to the hub genes. **B** A bi-partite network of the 46 hub genes and respective associated metabolites. Genes (blue) and metabolites (red) are represented as nodes, while the edges represent significant associations in our meta-analysis. Thickness of the edges corresponds to the explained variances of the associations. The type of line (dashed/dotted) represents the sign of the effect estimate (positive/negative). Only associations explaining at least 0.5% of the variance are shown

The majority of significantly associating transcripts (71.2%) are associated with more than one metabolite. As such, the top 1% of transcripts (83 genes) associating with the most metabolites were responsible for ~ 4.6% of the total associations representing association hubs (Fig. 2A). When focusing on the transcripts with more than 17 unique metabolite associations, we obtain a bi-partite network including transcripts of 46 genes as displayed in Fig. 2B. A large number of these association hub transcripts cluster around free carnitine (C0), acetylcarnitine (C2), the ratio of leucine|isoleucine/propionylcarnitine (Q19:(Leu|Ile)/C3), and the sum of acylcarnitines (AC-total). Visual inspection of the network reveals further interesting associations, e.g., the association of ATP Binding Cassette Subfamily A Member 1 (*ABCA1*) gene expression with proline and the proline-derived ratios glutamic acid/proline (Q17:Glu/Pro) and proline/ornithine (Q25:Pro/Orn). These associations suggest a potential cholesterol lowering effect of proline by *ABCA1* downregulation, previously observed for a phenylalanine-proline dipeptide [62].

### Comparability of whole blood and PBMC gene expression associations

We assessed the heterogeneity of association estimates obtained by meta-analyzing studies with gene expression from different tissues (whole blood in LIFE-Adult vs. PBMCs in LIFE-Heart AMI/non-AMI and the Sorb study) since this factor could not be accounted for in single study analyses but could be of biological relevance. The heterogeneity estimates (I^2^) of associations of the PBMC-only analysis are significantly smaller than those of the joint tissue analyses (paired one-sided Wilcoxon signed-rank test, *p* < 2.2 × 10^−16^). Distributions are shown in Supplemental Fig. S7, panel A–C. In the PBMC-only analysis, 83.8% of all associations have a heterogeneity estimate I^2^ ≤ 0.5, while in the main analysis including whole blood gene expression, 78.9% of all associations exhibit a heterogeneity estimate I^2^ ≤ 0.5. However, effect estimates of associations significant in at least one of the two analyses correlate highly across all metabolites (Pearson’s *ρ* = 0.96). In view of these observations, we decided to rely on the overall meta-analysis results.

### BMI mediation analysis reveals highly connected networks of transcripts and metabolites

We performed mediation analysis of effects of the potential causal chains: metabolite $\to$ gene expression $\to$ BMI (M $\to$ GE $\to$ BMI), i.e., the gene expression mediates metabolite effects on BMI, and alternatively, gene expression $\to$ metabolite $\to$ BMI (GE $\to$ M $\to$ BMI), i.e., metabolite levels mediate gene expression effects on BMI. Single study and meta-analyzed association statistics computed for testing mediation assumptions and for calculating mediation results are available as Online Supplemental Tables OS3 and OS4. In total, 33,204 GE-metabolite pairs comprising 65 metabolites and 8205 transcripts were considered in our mediation analysis, i.e., fulfilled our association requirements (see the “Material and methods” section). Of those, 676 transcript-metabolite pairs meet the requirements for testing for unidirectional mediation with GE as exposure only (GE $\to$ M $\to$ BMI). A total of 27,427 pairs were tested for unidirectional mediation with metabolites serving as exposure (M $\to$ GE $\to$ BMI) and 5101 pairs for testing for bi-directional mediation (i.e., 10,202 mediations tested). Thus, a total of 38,305 mediations were tested. Mediation summary statistics are provided at Online Supplemental Table OS5.

**Fig. 3:**
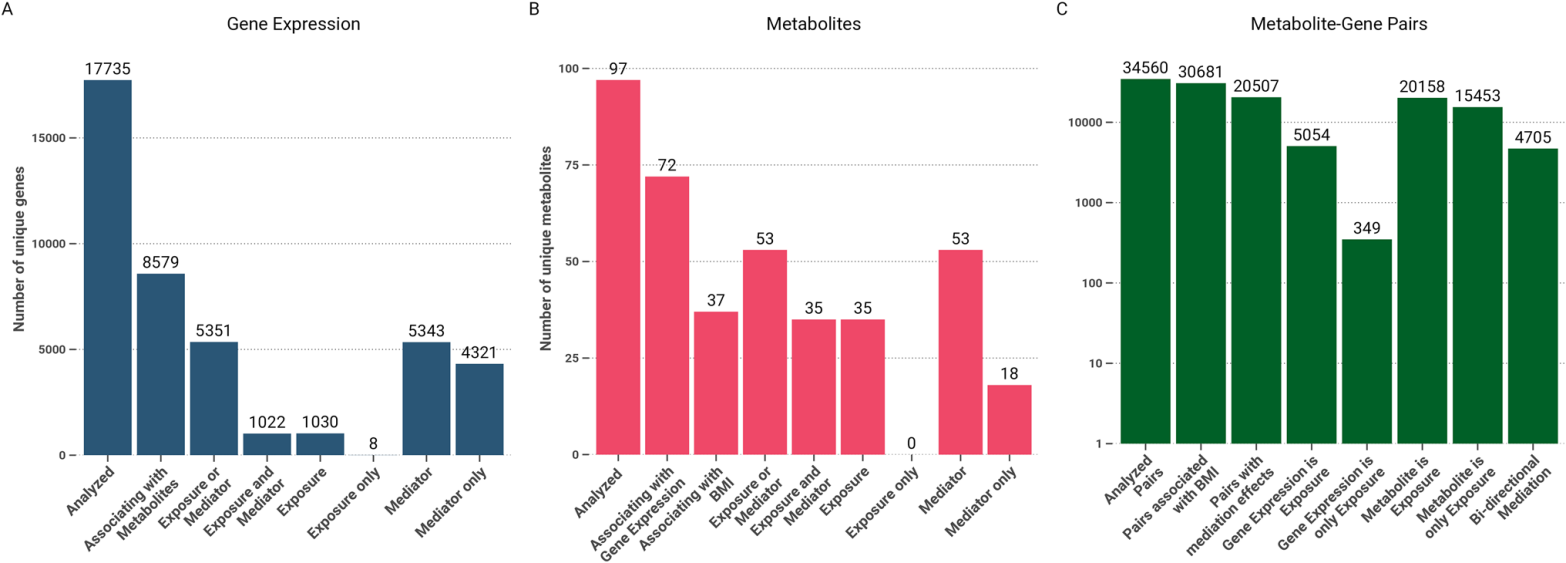
Number of unique transcripts, metabolites, and transcript-metabolite pairs that associate in the meta-analysis and are considered in mediation analyses towards BMI. **A** and **B** display count data for transcripts and metabolites analyzed in the association, as well as the mediation analysis, respectively. **C** displays information for associating and mediating unique transcript-metabolite pairs on a logarithmic scale. Notably, transcripts and metabolites are rarely involved in significant mediations exclusively as exposure

Correction of *p*-values on global and family level (the exposure is considered the family level) resulted in a total of 27,023 significant mediations (hierarchical FDR = 5%) involving 20,507 transcript-metabolite pairs. There, 375 mediations, corresponding to 349 transcript-metabolite pairs, were unidirectional with gene expression as exposures, only. Conversely, we found 15,453 transcript-metabolite pairs with unidirectional mediations with metabolites as exposure, only. Finally, 4705 transcript-metabolite pairs were bi-directional, i.e., were significant for mediations tested in both direction. An overview of available features and tested mediations is shown in Fig. 3. Notably, only eight transcripts and none of the metabolites were involved in significant mediations exclusively as exposures and not as mediators. The vast majority of 5351 transcripts involved in potential mediations were either both, mediator as well as exposure, or mediators only. Among the significant mediations on BMI, the five most frequently mediating or mediated metabolites, acetylcarnitine (C2), the sum total acylcarnitines (AC-total), 3-hydroxy-butyryl-carnitine (C4OH), octadecenoylcarnitine (C18:1), and propionylcarnitine (C3), were all metabolites involved in the oxidation of fatty acids. A summary of all mediations per metabolite is given in Supplemental Table S6. The five most frequently mediated or mediating transcripts were *BCL11A*, *ABCG1*, *FERMT3*, *NRCAM*, and *CCDC50*. As expected, the top mediated or mediating transcripts showed associations with multiple metabolites. In particular, this applies to *BCL11A*, *CCDC50*, and *ABCG1* which are also among the top five transcripts regarding number of metabolite associations (Supplemental Fig. S4). A summary of all mediations per transcript is provided in Supplemental Table S7.

We provide a network of the 250 largest mediation effects in Supplemental Fig. S8. A web application available at https://apps.health-atlas.de/mediation-net/ can be used to display networks of mediation effects on BMI for user-specified metabolites and transcripts. The displayed network can be further filtered based on the proportion of mediated effect sizes (PM). There, nodes represent individual metabolites and transcripts, and edges represent mediations. As an example, significant mediations involving the metabolite acetylcarnitine (C2) comprised 1402 transcripts. Filtering mediations with C2 based on a cutoff of PM ≥ 20% results in a more manageable network of strong mediations comprising nine transcripts. These include plausible genes such as the cholesterol efflux transporter *ABCG1* with a described role in the development of obesity, metabolic disease, and atherosclerotic lesions [64, 66].

### Metabolites more frequently serve as strong mediators

**Fig. 4:**
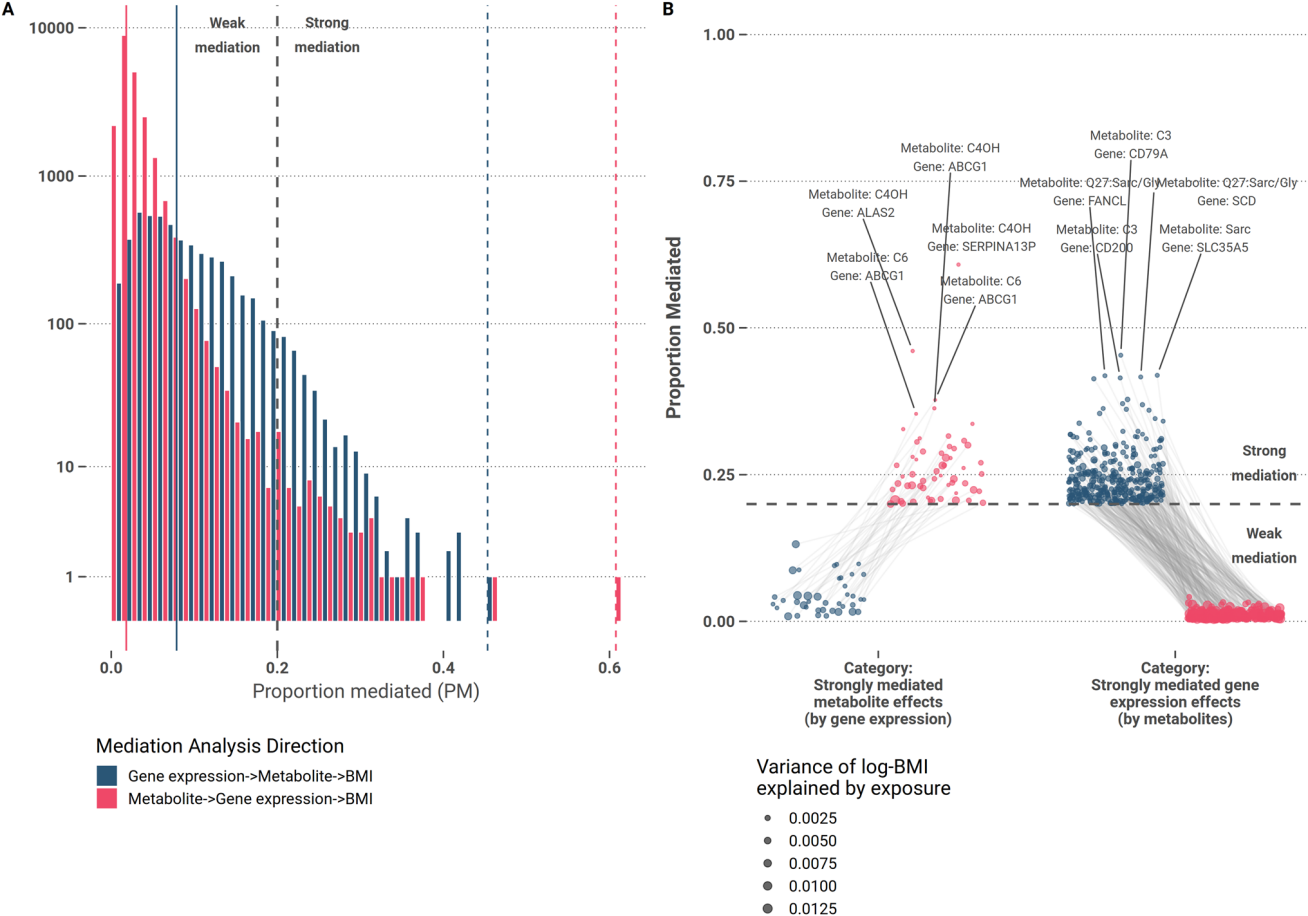
Analysis of mediation direction on BMI. **A** Distribution of mediated proportion of total effect for pairs of gene expression and metabolites. Only significant pairs were displayed. Blue = gene expression serves as exposure, red = metabolite serves as exposure. The solid lines mark the respective medians, the dashed colored lines the maxima, and the dashed grey line marks the threshold for a strong mediation effect, i.e., PM ≥ 20%, with a total of *n* = 403 pairs above this threshold. **B** Proportion of mediated effect size for strong mediations (PM ≥ 20%). PM of both mediation directions are compared. Blue dots = gene expression serves as exposure, red dots = metabolite serve as exposure, dots representing the same pair of gene expression, and metabolite are connected by grey lines. Dot sizes correspond to the variance of BMI explained by the exposure. Unconnected dots indicate a mediation that was only tested in one direction. The five strongest proportional mediation effects are annotated for each direction. Of note, no strong bi-directional effects were observed

To narrow down on interesting and biologically plausible mediations, we consider mediations with a high proportion of the mediated effect PM ≥ 20% and named them strong mediation effects. Among the 5352 significant mediations with gene expression as exposure, a total of 346 (6.5%) mediations were classified as “strongly mediated gene expression effects” (by metabolite effects). From the 21,671 mediations with metabolites as exposure, 57 (0.3%) were classified as “strongly mediated metabolite effects” (by gene expression effects). Mediations with high PM only occurred in one direction, i.e., even in cases of bi-directionally significant mediations, at most one of the mediation directions were classified as strong (Fig. 4B). The generally larger effects of BMI-metabolite associations were mediated frequently by gene expression effects, but to a smaller extent (M $\to$ GE $\to$ BMI: large *β*_mediation_, small PM, Supplemental Fig. S9). Conversely, the generally smaller effects of BMI gene expression associations were mediated to a larger extent by metabolites (GE $\to$ M $\to$ BMI: small *β*_mediation_, large PM, Fig. 4A, Supplemental Fig. S9). Statistics of pairs with strong mediation effect are provided at Online Supplemental Table OS5. Separating the strong mediations by their directions, the most frequent mediator genes were *AHSP*, *ABCG1*, *ALAS2*, *HBD*, and *CA1*. *AHSP*, *ALAS2*, and *HBD* are related to hemoglobin formation, and *CA1* codes for an erythrocyte-specific protein. Associations and mediations of these transcripts might be primarily attributed to additional blood parameters not adjusted for in the regression models. *ABCG1* codes for an important macrophage cholesterol efflux transporter, necessary for plasma HDL-C formation that has been extensively studied in connection to obesity and metabolic disease [70]. Effects of ABCG1 gene expression strongly mediate effects of various short-chain acylcarnitines (acetylcarnitine (C2), butyrylcarnitine (C4), 3-hydroxy-butyryl-carnitine (C4OH), isovalerylcarnitine (C5), hexanoylcarnitine (C6)), as well as of leucine|isoleucine (Leu|Ile) and hydroxyproline (OH-Prol).

Strong mediations can help to characterize the negative association of *ABCG1* with circulating Leu|Ile (*β* =  − 0.247, SE = 0.042, *p* = 2.83 × 10^−09^), which was also reported by Bartel et al. [20]. Both *ABCG1* gene expression (*β* =  − 0.035, SE = 0.005, *p* = 4.95 × 10^−12^) and Leu|Ile levels (*β* = 0.037, SE = 0.010, *p* = 1.28 × 10^−4^) are associated with BMI in our data and show strong mediation of the metabolite effect via gene expression (*β*_mediation_ = 0.008, CI95% = [0.005, 0.011], *p* = 2.06 × 10^−10^, PM = 0.21). Bartel et al. discuss potential co-regulation between BCAAs and the cholesterol metabolism at the transcriptional level including ABCG1 gene expression and respective implications for obesity associations [20].

### Mediations provide mechanistic insights for genetically regulated obesity genes

**Fig. 5:**
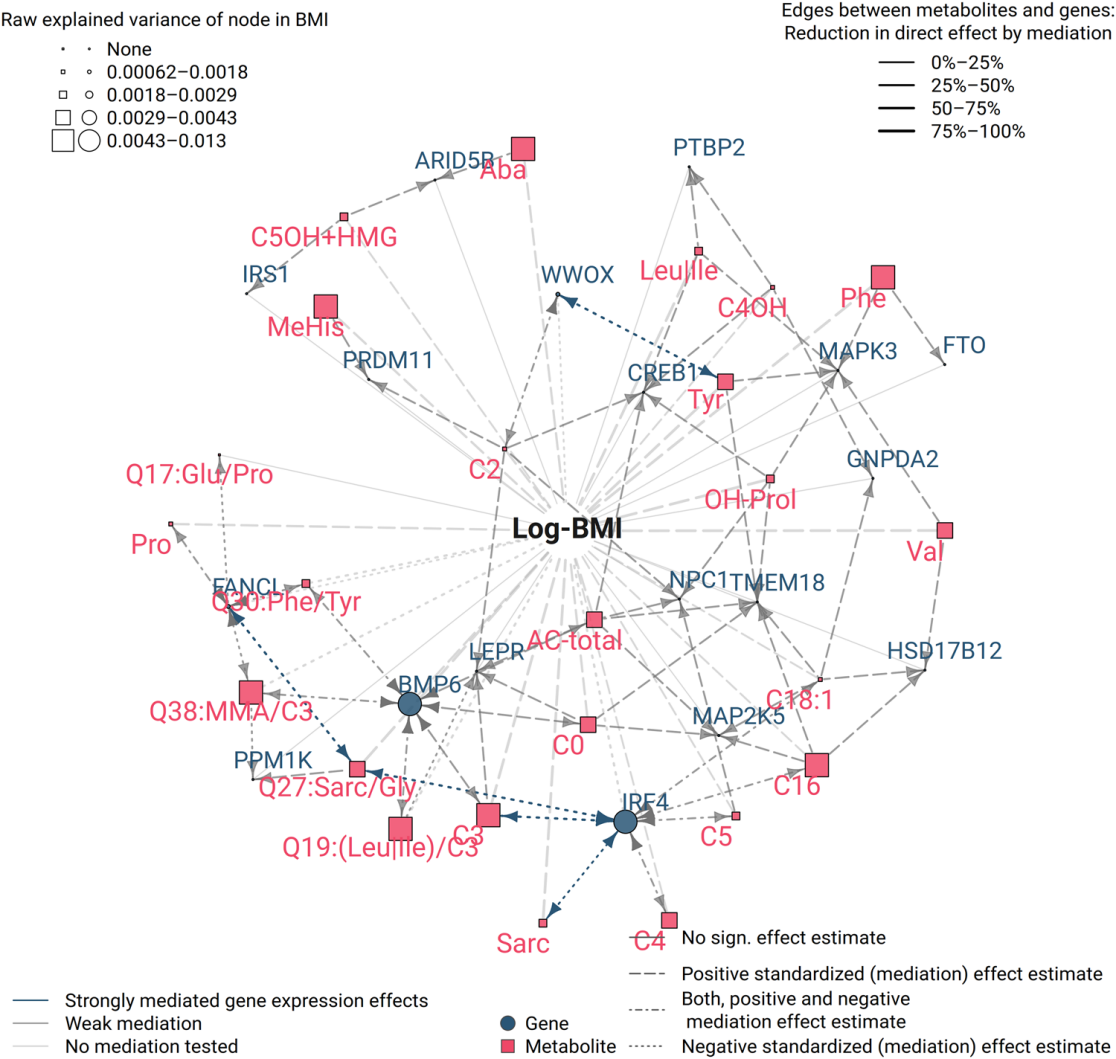
Mediation network involving expressions of genes genetically associated with BMI nodes represents genes and metabolites acting as exposure or mediator of BMI effects. Colors represent the class of the nodes: Blue = gene expressions, red = metabolites. Arrows between metabolite and gene expression nodes represent the direction of mediation, either uni- or bi-directional. The line color depicts the strength of the mediation by the classification “strongly mediated metabolite effects” (red), “strongly mediated gene expression effects” (blue), or “weak mediations” (gray). The line thickness represents the size of the indirect effect relative to the direct effect. The size of the node indicates variance of BMI explained by each node in univariate association analysis. Edges between nodes indicate significant mediations

In this section, we focus on genes reported for genetic associations with BMI. As an example, application of our catalogue of mediation effects, we provide additional functional evidence underlying these associations. We retrieved 51 such genes from Srivastava et al. [61]. Additionally, we included one gene that was both genetically associated to metabolites (BCAA) and T2D, namely *PPM1K* [60]. In our meta-analysis, we detected 87 associated metabolite-transcript pairs of 27 catalogue genes with 32 metabolites. Of these associating pairs, 80 qualified for mediation analysis, i.e., at least one of the participating features showed an association with BMI. The pairs comprised 25 of the originally identified genetically regulated obesity genes. Figure 5 displays all 93 significant mediations, involving 18 transcripts and 24 metabolites (Supplemental Table S8). Of these mediations, five, involving *IRF4*, *FANCL*, and *WWOX*, were classified as “strongly mediated gene expression effects” (Table 2). For instance, *IFR4* was mediated by propionylcarnitine (C3), sarcosine, and the ratio of sarcosine/glycine (Q27). *IRF4* codes for the interferon regulatory factor 4 and has a diverse role as repressor of adipogenesis and as a negative regulator of the inflammatory response to obesity through M2 macrophage polarization [71]. Experiments with adipocyte-specific Irf4^−/−^ knock outs in mice showed associations with increased weight gain and insulin resistance. The estimated total effect of *IRF4* gene expression on BMI (*β*_total_ =  − 0.01) was mediated with a PM = 0.24 by sarcosine, with a PM = 0.26 by Q27:Sarc/Gly and with a PM = 0.25 by C3 in our data. C3 is related to BCAA metabolism and oxidation of odd-chain fatty acids, as well as the glycine metabolism, for which sarcosine is a precursor. C3 is also closely linked to insulin sensitivity playing a role in obesity-related disease [72, 73]. Additionally, *WWOX* has been investigated in its role in metabolic disorders such as insulin resistance [74].

**Table 2:** Strong mediations (proportion of mediated effect, PM ≥ 0.2) of effects involving transcripts of genes associated with BMI

**Exposure (gene)**	**Exposure (probe)**	**Mediator (metabolite)**	**Mediator (abbreviation)**	**Mediation effect size**	**CI95% (lower)**	**CI95% (upper)**	***p***-**value**	**Local FDR**	**Global FDR**	**Proportion mediated**	**Total effect on BMI**
**FANCL**	ILMN_1754045	Sarcosine/glycine	Q27:Sarc/Gly	−0.003	−0.0047	−0.0016	3.5E−16	1.7E−15	1.2E−14	0.42	−0.0072
**IRF4**	ILMN_1754507	Sarcosine/glycine	Q27:Sarc/Gly	−0.0027	−0.0045	−0.0012	4.9E−11	8.6E−11	1.4E−19	0.27	−0.01
**IRF4**	ILMN_1754507	Propionyl-carnitine	C3	−0.0027	−0.004	−0.0015	1E−21	7.3E−21	1.4E−19	0.25	−0.011
**WWOX**	ILMN_2415776	Tyrosine	Tyr	−0.0015	−0.0025	−0.00062	1.3E−10	2.5E−10	6.4E−10	0.25	−0.0058
**IRF4**	ILMN_1754507	Sarcosine	Sarc	−0.0024	−0.0043	−0.00095	8.5E−09	9.9E−09	1.4E−19	0.24	−0.01

## Discussion

In this study, we performed a genome-wide gene expression/metabolome association meta-analysis of three cohorts with up to 7706 subjects using the same gene expression and metabolome technology across studies. We further characterized the relationships of individual transcripts and metabolites by analyzing mediation of gene expression or metabolite effects towards BMI. In our meta-analysis, we identified 37,461 significant associations including 72 metabolites and metabolic ratios and gene expression levels of 8579 genes. Previous association studies of blood gene expression and circulating metabolites were limited in sample size finding up to 1400 associations [20, 21]. Thus, we significantly extended the catalogue of expression/metabolite associations.

Furthermore, we found that 20,507 pairs of 5351 transcripts and 53 metabolites showed third-variable effects that present potential mediations towards BMI in 27,023 instances considering the two mediation pathways M → GE → BMI and GE → M → BMI. Of those mediations, 21,671 followed the first mediation pathway, while 5352 followed the second one. Four thousand seven hundred five transcript-metabolite pairs showed potential bi-directional mediation of effects. Transcripts of the genes *BCL11A*, *ABCG1*, *FERMT3*, *NRCAM*, and *CCDC50* were the most frequently mediated or mediating transcripts. ATP binding cassette subfamily G member 1 (*ABCG1*) encodes a cholesterol efflux transporter in macrophages and monocytes. Impaired monocyte cholesterol efflux in type 2 diabetics was linked to *ABCG1* gene expression [63]. The overall potential role of *ABCG1* and reduced cholesterol efflux in adipogenesis and its wider relevance in cardiometabolic disease and atherosclerotic lesions has been extensively studied [64–66]. The kindlin-3 protein is a product of the *FERMT3* gene, which, mostly expressed in hematopoietic cells, has a function in hemostasis regulation [67]. *NRCAM* codes for a cell adhesion molecule that was previously reported downregulated in aged CD8^+^ T cells and investigated for its role in immunosuppression [68, 69]. We classified 403 potential mediations as strong by a PM cutoff of 0.2. According to this definition, 346 strong mediations of gene expression effects (by metabolite effects, i.e., GE → M → BMI) and 57 strong mediations of metabolite effects (by gene expression effects, i.e., M → GE → BMI) were detected. Of note, we found no strong bi-directional mediations, i.e., strong mediations clearly favored one of the mediation directions. Thus, strong mediations represent clear candidates of unidirectional causal effects by not only increasing confidence in the effect estimates but also allowing assigning a direction of causality.

Several transcripts formed association hubs, associating with up to 28 metabolites (Fig. 2). The five most broadly associating transcripts, *BCL11A* (28 metabolites), *CCDC50* (25 metabolites), *ABCG1* (24 metabolites), *PCCA* (23 metabolites), and *ACADVL* (22 metabolites), cover a variety of functions of blood cell differentiation and composition, immune function, fatty acid oxidation, and cholesterol transport. The top association hub was the transcript of *BCL11A*, coding for the fetal γ-globin silencing factor protein “B-cell lymphoma/leukemia 11A.” The protein controls hemoglobin switching during maturation by repressing expression of γ-globin [75]. Elevated expression of *BCL11A* in human beta cells was reported to be negatively correlated to insulin secretion, contributing to an increased risk of type 2 diabetes development, making this gene a prominent target for further studies [76–79]. Induction of γ-globin synthesis was previously demonstrated by administration of short-chain fatty acids and derivatives, specifically the four-carbon butyrate and the 2-carbon acetate [80, 81]. In our data, *BCL11A* gene expression was negatively associated with BMI and a range of acylcarnitines (C2, C3, C4OH, and total acylcarnitines). In addition, we observed mediations of its effects on BMI by short-, medium-, and long-chain acylcarnitines as well as free carnitine (C0). This observation is consistent with the results reported by Pace et al. and Liakopoulou et al., making *BCL11A* a potential missing link in this mechanism, i.e., mediation of metabolite effects on BMI by *BCL11A* gene expression [80, 81]. We propose that increased levels of fatty acids decrease expression of *BCL11A* promoting γ-globin expression. The subsequently induced synthesis of fetal hemoglobin affects BMI or BMI-associated phenotypes such as type 2 diabetes and insulin secretion.

Our analysis was aimed at discovering mediations of effects towards BMI. Principally, the assumed causal order cannot be proven with statistical methods of mediation analysis but requires prior knowledge or interventional experimental designs [56]. When testing for mediation in a prescribed pathway, e.g., M $\to$ GE $\to$ BMI, one essentially exploits the correlation structure between the three variables M, GE, and BMI. However, this correlation structure can also be produced by alternative causal relationships, in the above case, for example, by the alternative chain BMI $\to$ GE $\to$ M [82]. Fairchild and McDaniel recommend to combine the statistical evidence from mediation analysis with biological information to infer the underlying causality [56]. Our results need to be considered in the light of this limitation. Indeed, in some instances, there is evidence that the causal chains analyzed here are reversed, i.e., BMI serves as exposure rather than outcome. Obesity can change the cellular composition of adipose tissue, accompanied by an increased infiltration by macrophages and other immune cells, which is linked to increased cytokine secretion, predominantly TNF-α, as well as a changed T-cell population, leading to low-grade and chronic inflammation [83, 84]. For example, among obesity genes reported in literature, *IRF4* has a pronounced role in obesity-induced inflammation, supporting the BMI $\to$ GE causality [71]. Another example of a potentially inverse causal order (BMI $\to$ GE $\to$ M) is mediations involving the transcript abundance of *ABCG1*. Previous studies showed that *ABCG1* suppression in macrophages increased cytokine levels, biased macrophage polarization towards pro-inflammatory M1 macrophages, and was a cause of foam cell formation that accelerated atherosclerosis [64, 85–87]. While mediation results suggest that ABCG1 gene expression is a strong mediator of nine BMI-metabolite associations, the reverse causality of BMI $\to$ GE is supported by previous research. Specifically, the impact of adipokines on macrophage polarization and, subsequently, *ABCG1* gene expression differences in M1 and M2 macrophages has been previously reported [88].

An example where previous research supports the tested mediation direction involves *BMP6* (coding for the bone morphogenetic protein 6) gene expression, where increased *BMP6* activity induces the transcription factor PPARγ in adipocytes, leading to enhanced glucose uptake and has therefore been indicated as a drug target to treat insulin resistance [89]. We observed mediation effects of *BMP6* via free carnitine (C0) and various acylcarnitines such as propionylcarnitine (C3). *BMP*’s documented effect on glucose uptake, and subsequently, the glycolysis rate also affects the fatty acid metabolism, making the observed mediation direction GE $\to$ M $\to$ BMI plausible.

Our study has several limitations. First, we focused on the link between gene expression and metabolites which is understudied in comparison to, e.g., genetic associations of gene expression or metabolite features. Our mediation results need to be considered with caution because a causality cannot be proven by this type of analysis, in particular with respect to the direction of the causal path as discussed above. Additionally, due to the complexity of omics interaction, the presence of unmeasured confounding cannot be fully excluded in our analyses. This might explain some of the bi-directional mediation effects observed. On the other hand, strong mediations appear to show a clear direction preference increasing confidence in these findings. In summary, while our analysis approach provides a necessary step to establish causal chains, further experimental approaches are required to validate these findings. As a major application of our catalogue, we considered BMI as an endpoint due to its wide availability and extensive available research although this phenotype is only a proxy to metabolic diseases. The available metabolite panel, limited to amino acids and acylcarnitines, represented only a small part of the human metabolome in one tissue with limited generalizability. However, the available metabolites were all fully characterized, and the measurement methodology is highly standardized, minimizing variability attributable to technical sources [30, 31]. We demonstrate in a previous study [32] that our metabolite panel is affected by fasting status, which differed between studies, i.e., participants of LIFE-Adult and the Sorbs study were at fasting, while participants of LIFE-Heart were not. We adjusted our analyses for fasting hours, but this information was not available for the Sorbs study contributing to the heterogeneity of results. Lastly, the usefulness of BMI as a risk factor for metabolic diseases has long been debated. More meaningful classification were proposed, e.g., by measuring perturbations of the metabolism and distinguishing between metabolically healthy and unhealthy obesity [90]. Additionally, by not adjusting for T2D in the association analysis, mediation results on BMI might comprise those attributable to T2D or other BMI-associated metabolic traits.

In conclusion, by our study, we considerably expand human circulating blood transcriptome-metabolome associations using the same phenotyping platforms in three large cohorts. We further annotated this catalogue by a mediation analysis investigating the impact of these omics layers on BMI. This analysis provides both plausible transcript-metabolite pairs whose association to BMI warrants further functional investigation and a broader impression of the interplay between metabolome and transcriptome in relation to a common phenotype such as BMI, as well.

### Supplementary Information

Below is the link to the electronic supplementary material.

Supplementary file1 (DOCX 8.10 MB)

Supplementary file2 (XLSX 2145 KB)

Supplementary file1 (DOCX 11.3 KB)

## Data Availability

All single study and meta-analyzed summary statistics are publicly available online as Online Supplemental Tables OS1-5 at 10.5281/zenodo.7104774.
