# Supplementary material for: An atlas of genome-wide gene expression and metabolite associations and possible mediation effects towards body mass index
Source: J Mol Med (Berl). 2023 Sep 6;101(10):1305–21. doi: 10.1007/s00109-023-02362-z (PMC10560167; doi:10.1007/s00109-023-02362-z)
Supplement: Supplementary file 1 — Supplementary file1 (DOCX 8.10 MB) [file 109_2023_2362_MOESM1_ESM.docx]

Supplemental Data - An atlas of genome-wide gene expression and metabolite associations and possible mediation effects towards body mass index

Carl Beuchel^1^, Julia Dittrich^2^, Susen Becker^2,3^, Holger Kirsten^1,4^, Anke Tönjes^5^, Peter Kovacs^7^, Michael Stumvoll^5^, Markus Loeffler^1,4^, Andrej Teren^6^, Joachim Thiery^2,4^, Berend Isermann^2,4^, Uta Ceglarek* ^2,4^ and Markus Scholz* ^1,4^

^1^ Institute for Medical Informatics, Statistics and Epidemiology, Leipzig University, Leipzig, Germany

^2^ Institute of Laboratory Medicine, Clinical Chemistry and Molecular Diagnostics, Leipzig University, Leipzig, Germany

^3^ Institute of Legal Medicine, Department of Forensic Toxicology, University Leipzig, Germany

^4^ LIFE – Leipzig Research Center for Civilization Diseases, Leipzig University, Leipzig, Germany

^5^ Medical Department III – Endocrinology, Nephrology, Rheumatology, University Hospital Leipzig, Leipzig, Germany

^6^ Heart Center Leipzig, Leipzig, Germany

^7^ Deutsches Zentrum für Diabetesforschung, Neuherberg, Germany

*equal contribution

[1 Supplemental Methods 3](#_Toc118194232)

[1.1 Gene expression sample collection, pre-treatment and measurement 3](#_Toc118194233)

[1.1.1 Sample collection in LIFE Adult 3](#_Toc118194234)

[1.1.2 Sample collection in LIFE Heart 3](#_Toc118194235)

[1.1.3 Sample collection in the Sorbs study 3](#_Toc118194236)

[1.2 Gene expression data pre-processing 4](#_Toc118194237)

[2 Supplemental Figures 6](#_Toc118194238)

[2.1 Supplemental Figure S1 6](#_Toc118194239)

[2.2 Supplemental Figure S2 7](#_Toc118194240)

[2.3 Supplemental Figure S3 8](#_Toc118194241)

[2.4 Supplemental Figure S4 9](#_Toc118194242)

[2.5 Supplemental Figure S5 10](#_Toc118194243)

[2.6 Supplemental Figure S6 11](#_Toc118194244)

[2.7 Supplemental Figure S7 12](#_Toc118194245)

[2.8 Supplemental Figure S8 13](#_Toc118194246)

[2.9 Supplemental Figure S9 14](#_Toc118194247)

[3 References 15](#_Toc118194248)

# Supplemental Methods

## Gene expression sample collection, pre-treatment and measurement

### Sample collection in LIFE Adult

In the LIFE-Adult study, 3,173 whole blood samples were collected in Tempus Blood RNA Tubes (Life Technologies) and stored at -80˚C. Processing and hybridizing of isolated RNA to Illumina HT12 v4 Expression BeadChips (Illumina, San Diego, CA, USA) in batches of 48 was followed by measurement on Illumina HiScan (Holdt et al. 2013). Illumina GenomeStudio (without additional background correction) was used to extract raw data of 47,231 gene-expression probes (and 876 control probes).

### Sample collection in LIFE Heart

For LIFE-Heart AMI/non-AMI participants, 2,856 (non-AMI) respectively 1,287 (AMI) samples of peripheral blood mononuclear cells (PBMCs) were isolated using Cell Preparation Tubes (CPT, Becton Dickinson), respectively. Details of sample preparation are described elsewhere (Holdt et al. 2010). TRIzol reagent (Invitrogen) was used for total RNA extraction and a UV-Vis spectrophotometer (NanoDrop, Thermo Fisher) for RNA quantification. For probe synthesis, samples were ethanol precipitated using GlycoBlue (Invitrogen) and then diluted to 50–300 ng/μl per sample. Hybridisation and measurement followed the same procedure as in the LIFE-Adult study.

### Sample collection in the Sorbs study

Blood samples from 988 Sorb study participants were collected in VACUTAINER CPT (Cell Preparation Tubes). The TRIzol protocol (Thermo Fisher Scientific) was used for RNA extraction from extracted PBMCs. DNAse I digestion and RNA clean-up was carried out using RNeasy MinElute Cleanup Kit (Qiagen, Hilden, Germany). Agilent 2100 Bioanalyzer (Agilent Technologies, Palo Alto, CA, USA) using the RNA 6.000 LabChip Kit (Agilent Technologies) was used according to the manufacturer’s instructions to examine RNA integrity and concentration. Reverse transcription to cDNA of 250ng RNA was carried out using the Target Amp labelling kit (Illumina, SanDiego, CA, USA and Superscript III, Life Technologies, Gaithersburg, MD, USA). Superscript III (Life Technologies, Gaithersburg, MD) and Target Amp labelling kit (Illumina, SanDiego, CA, USA) were used to synthesize cRNA by in vitro transcription. The RNeasy kit (QIAGEN, Hilden, Germany) was used to remove unincorporated nucleotides. The cRNA was hybridized to the Illumina Human HT-12 v4 Expression Bead Chip. The arrays were scanned using an Illumina High Scan SQ.

## Gene expression data pre-processing

Data of LIFE-Adult, LIFE-Heart (distinguished between AMI and non-AMI) and the Sorbs study were all preprocessed using the same procedure.

Initially, expression values were log2-transformed and quantile normalized (Schmid et al. 2010). Next, removal of technical batch effects was facilitated using an empirical Bayes method as implemented in the ‘ComBat’ function of the ‘sva’ R package with added covariates to preserve these effects (Johnson et al. 2007; Leek et al. 2012). ComBat allows for adding of covariates, whose effects are to be preserved during the batch-adjustment. Thus, the phenotypes of interest log-BMI and diabetes status, as well as covariates age, sex, hematocrit, monocytes, neutrophils and hours fasted (not in the Sorb cohort, for the information was unavailable) were added in the model to preserve the effects for the subsequent association analysis. The expression chip-ID of a sample was used as batch variable. Technical requirements of the method were at least two samples per batch. This led to exclusion of one (0.1%) sample in the Sorbs study and two (0.05%) samples in the LIFE-Heart study. No such exclusions were necessary in the LIFE-Adult study.

Gene expression probes were excluded when not expressed in more than 5% of samples and when they significantly associated with batches (after Bonferroni correction) after the batch-adjustment. Low-quality samples were removed based on three steps. Samples with a number of detected genes ±3 × interquartile ranges (IQR) from the median were removed. For each sample, a filter based on the Mahalanobis distance of the gene-expression of control-probes to an averaged sample was imposed, including biotin control-probes, signal of low-concentration control probes, signal of medium-concentration control probes, signal of mismatch control probes, signal of negative control probes and signal of perfect-match control probes) of each sample (Cohen Freue et al. 2007). Samples above +4×IQR of the median were removed. Finally, samples with an Euclidean distance of their expression values +4×IQR from the median sample were removed (Schmid et al. 2010). Gene expression filter steps are summarized in **Supplemental Table 9.** Preprocessing was facilitated using the pipeline implemented in the R package ‘HT12ProcessoR’.

Mapping of gene expression probes to unique genes was conducted via the Ingenuity Pathway Analysis database (QIAGEN Inc., accessed on 2019-04-04). 36,367 gene expression probes were successfully mapped to a single genomic region within a margin of 1Mb of the location of the respective gene in the human genome (hg19). This resulted in 36,367 probes being mapped to 22,645 unique genes.

# Supplemental Figures

## Supplemental Figure S1


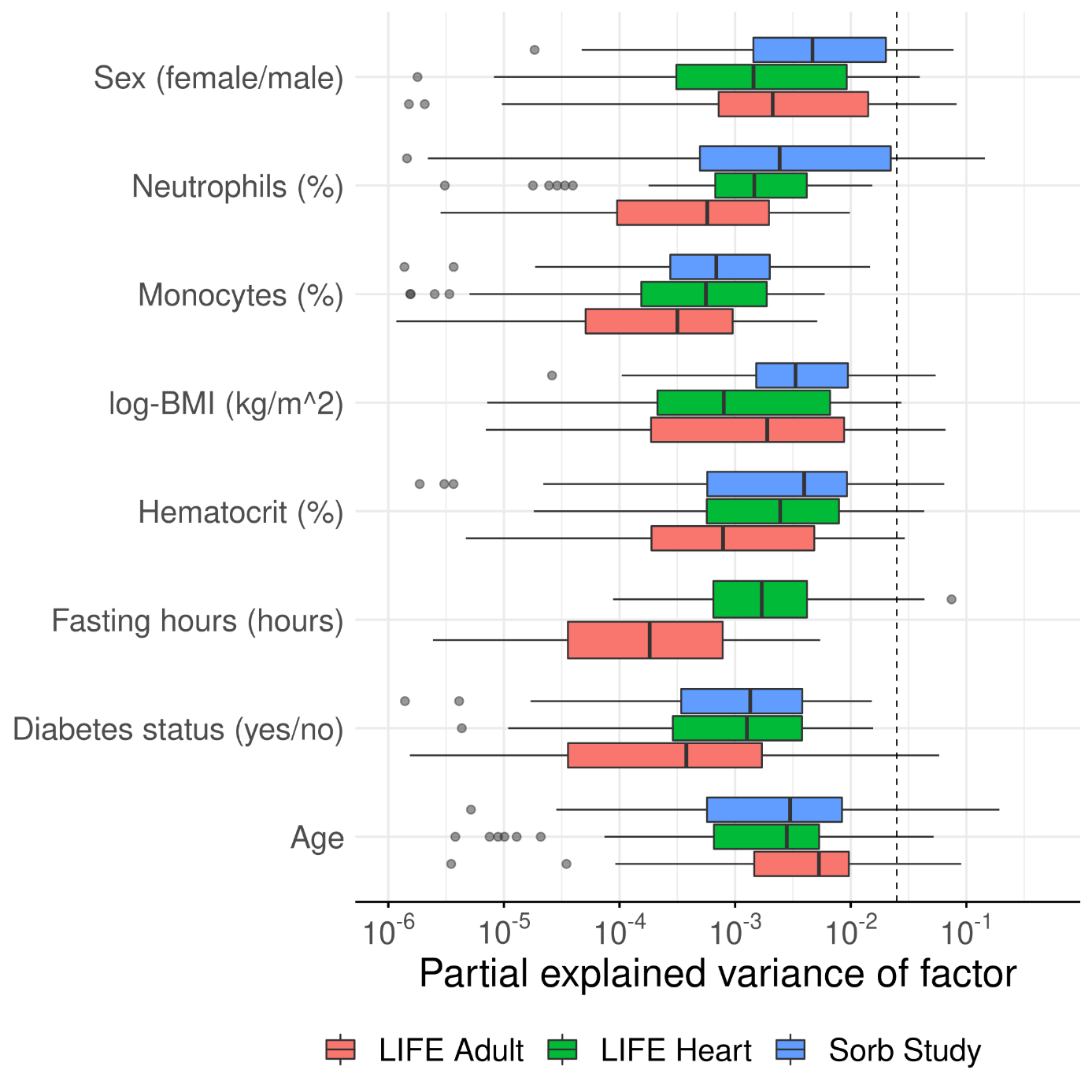


Supplemental Figure 1 Distributions of the partial explained variances of selected confounders in metabolites separately for the studies LIFE-Adult, LIFE-Heart (including AMI) and the Sorbs study. Boxplots represent the distribution of partial explained variance of respective factors (y-axis) for the 97 analyzed metabolites and quotients.

## Supplemental Figure S2


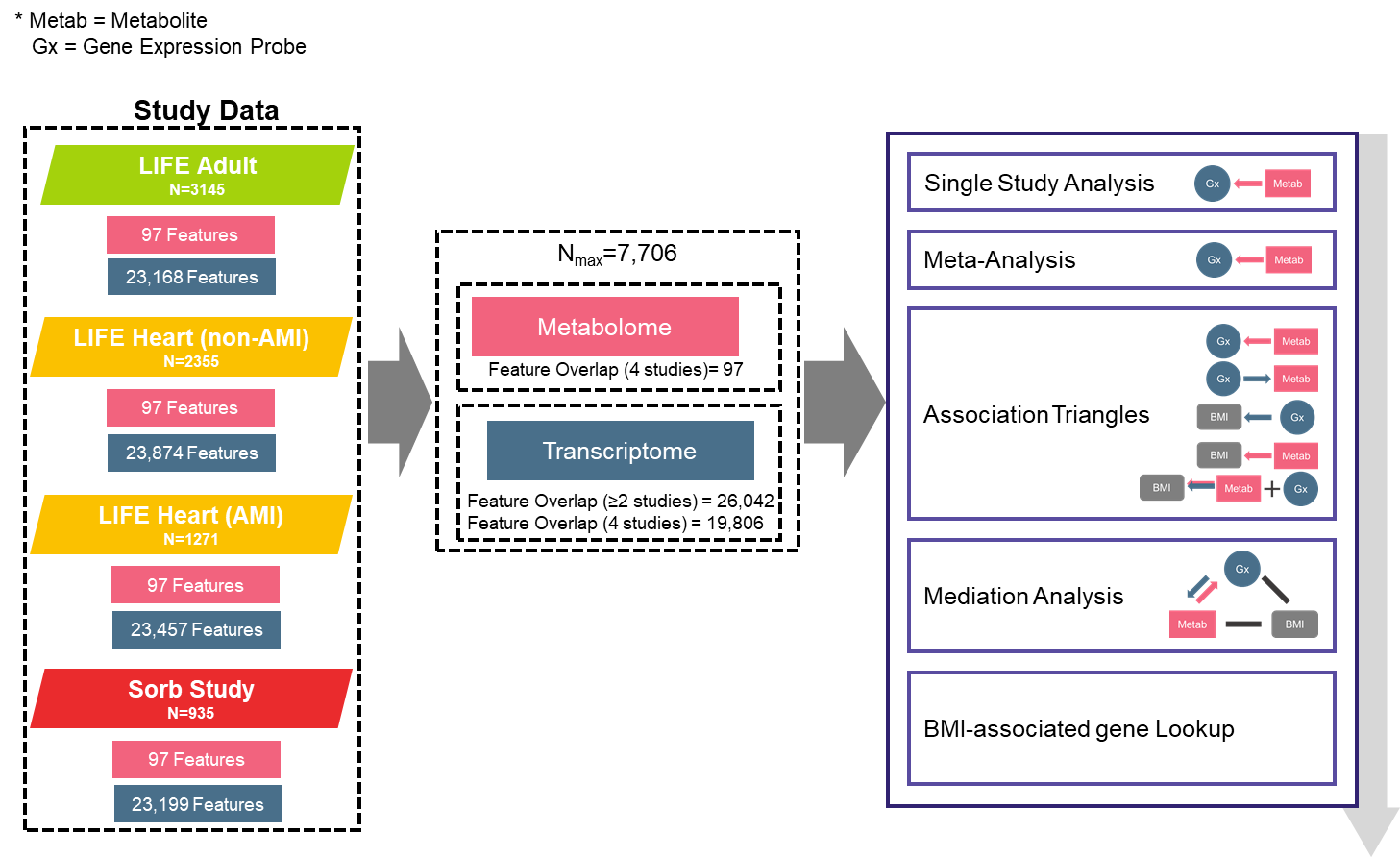


Supplemental Figure 2 Overview of the data available and the analyses conducted in the present study. Sample size of matched metabolite and gene expression data in each study as well as total sample size and feature overlap (for meta-analysis, a feature has to be available in at least two studies) is provided.

## Supplemental Figure S3


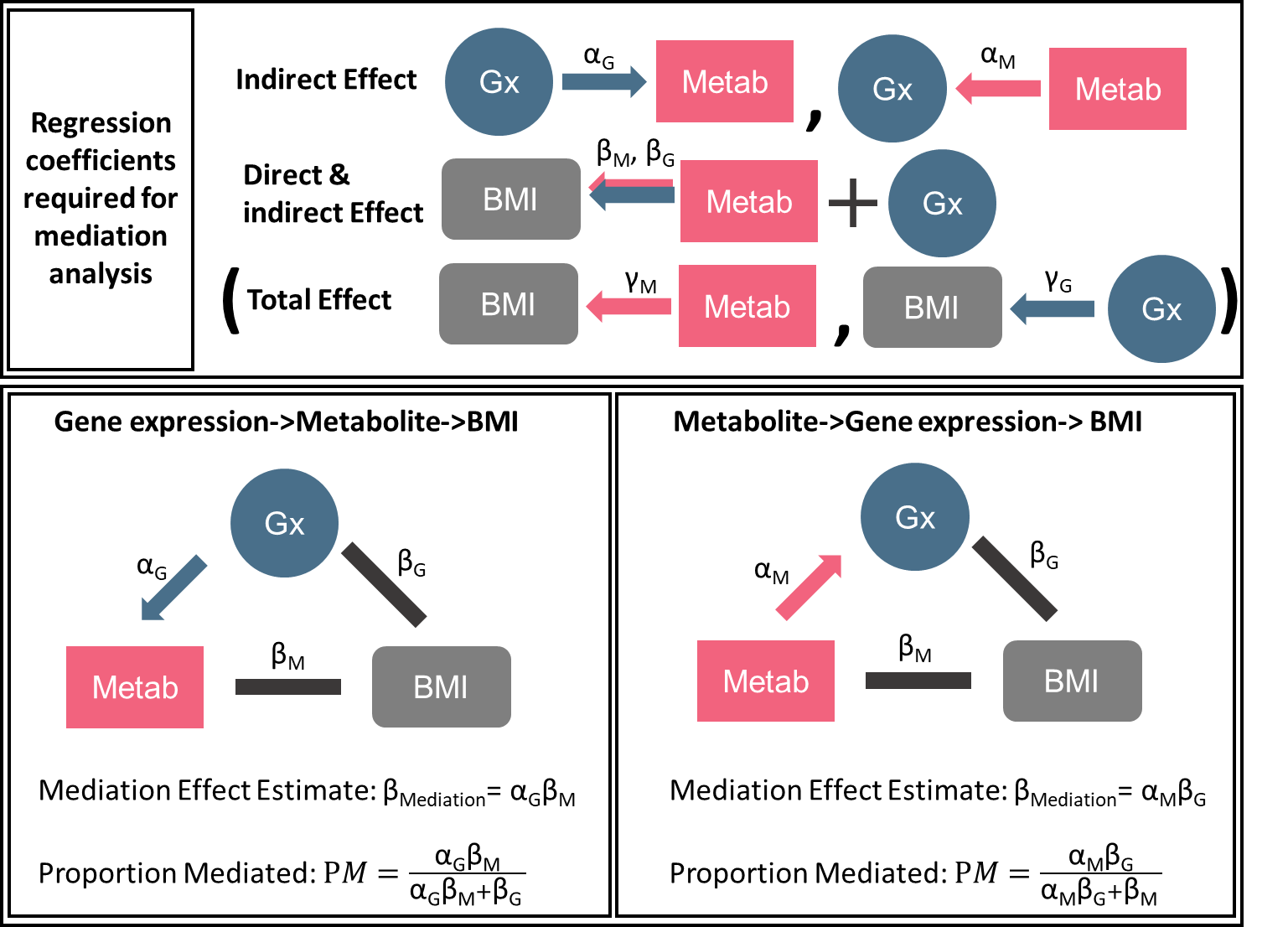


Supplemental Figure 3 Overview of the notation used and regression models needed for mediation analysis. In the upper panel, regressions necessary for calculation of the direct, indirect and total effect are listed and correspond to respective regression coefficients. In the lower left panel, formulas for computation of the mediation effect estimate and the proportion mediated (PM), i.e. the fraction of the total effect of the exposure on the outcome (BMI) being mediated are provided for mediations with gene-expression as exposure and metabolites as mediator. In the lower right panel, the same is shown for mediations of metabolite effects on BMI via gene expression effects.

## Supplemental Figure S4


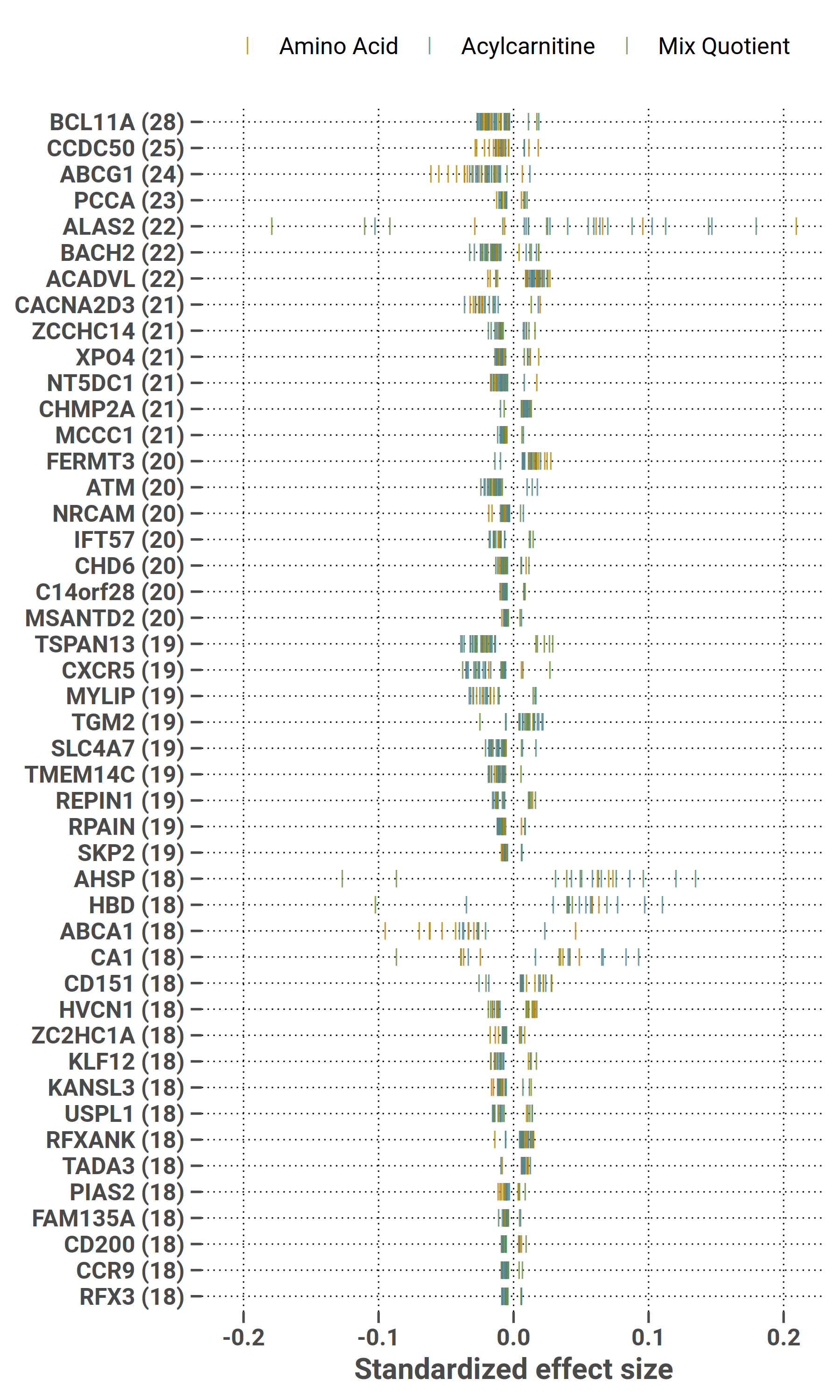


Supplemental Figure 4 Effect estimates of the 50 most frequently associated genes in the gene expression-metabolite association meta-analysis. For each association, the standardized effect estimate is shown and the classes of the associated metabolites are color-coded. Genes are sorted by the number of associations from top to bottom. The number of uniquely associating metabolites per gene are given in parenthesis after the gene names.

## Supplemental Figure S5


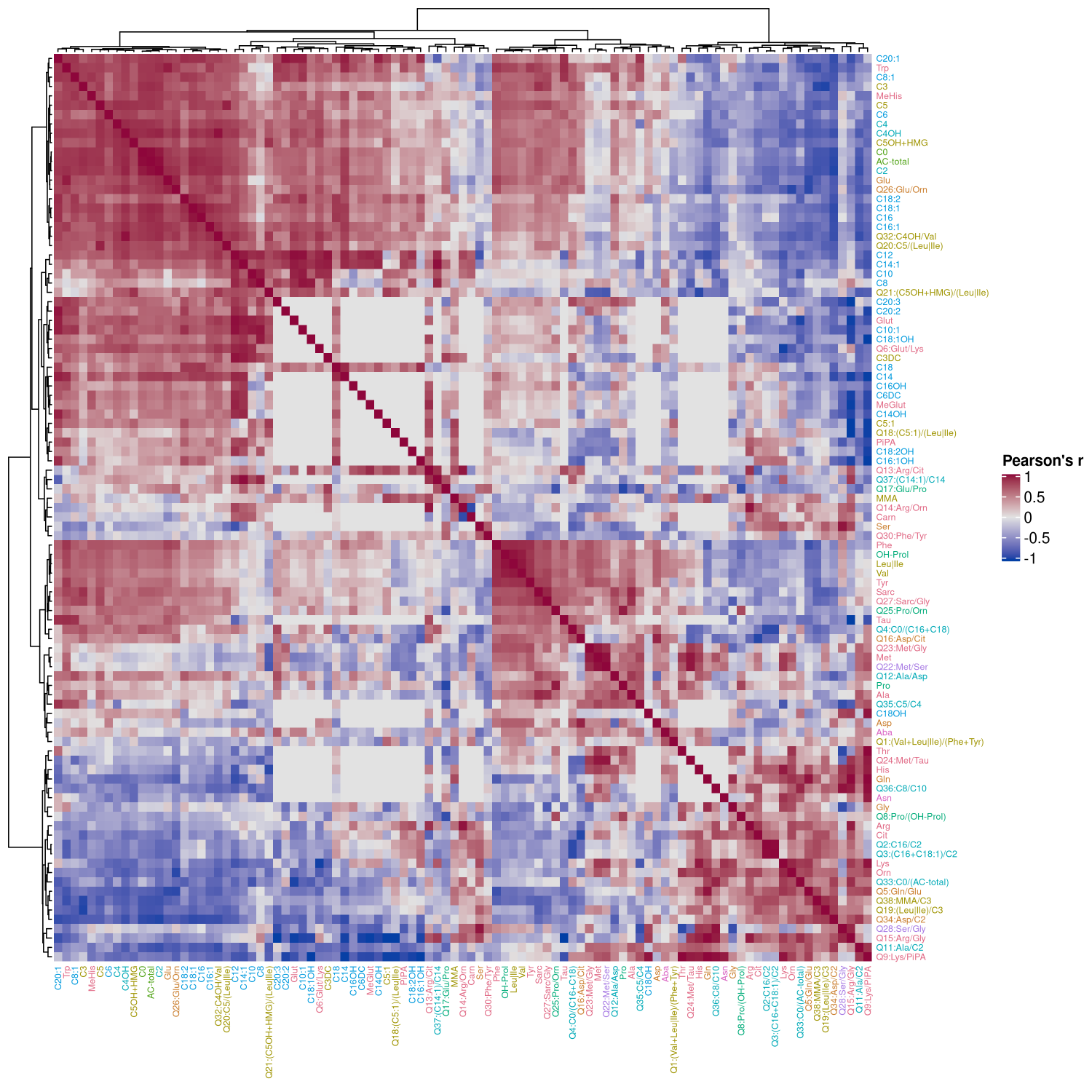


Supplemental Figure 5 Pairwise Pearson’s correlation coefficients of effect estimates of the gene-expression metabolite associations. Only coefficients significant for at least one of the paired metabolites were used for correlation analysis. Hierarchical clustering was applied using the ‘hclust’ function with the ‘ward.D2’ method based on Euclidean distance.

## Supplemental Figure S6


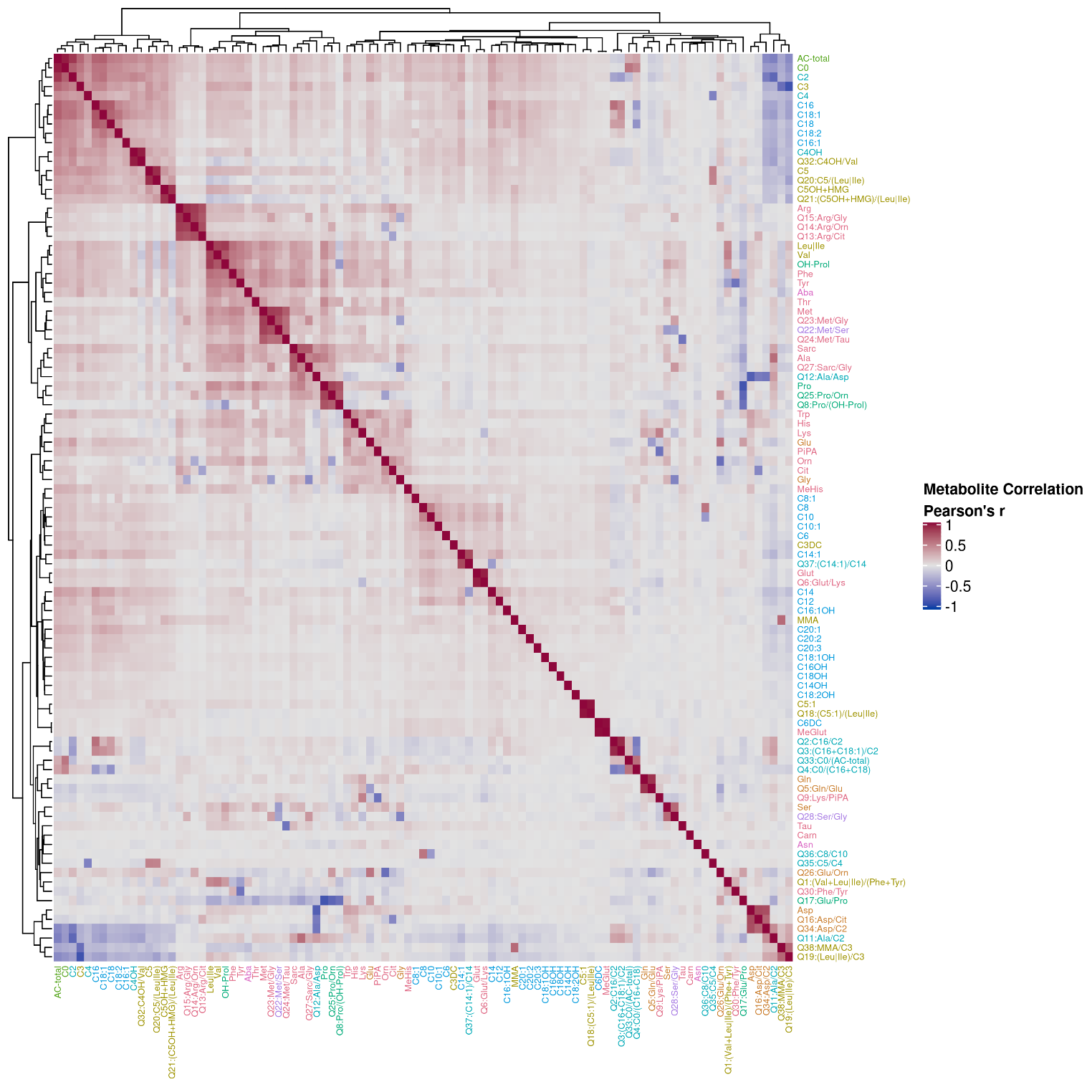


Supplemental Figure 6 Pearson’s pairwise correlation coefficient of metabolite levels of all pairs of the 97 metabolites considered in this study. Metabolite measurements of the available studies were pooled and correlated jointly. Coloration of metabolite names represents pathway membership. Hierarchical clustering was applied using the ‘hclust’ function with the ‘ward.D2’ method based on Euclidean distance.

## Supplemental Figure S7

E

D

C

A

B


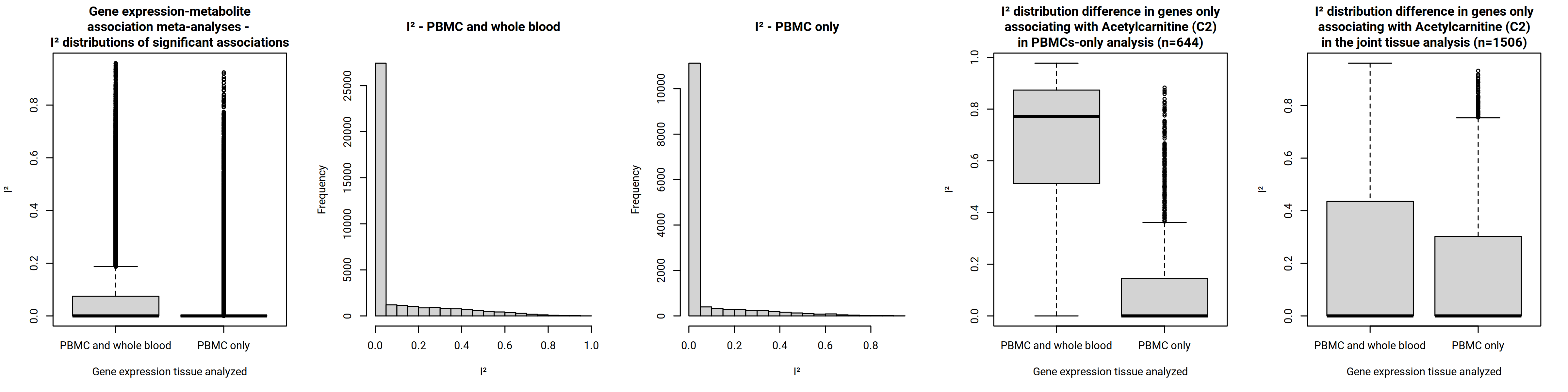


Supplemental Figure 7 Distributions of heterogeneity estimates from a gene expression-metabolite association meta-analysis using a random effects model. In LIFE-Adult, gene expression measurements were available from full blood and from peripheral blood mononuclear cells (PBMCs) in LIFE-Heart and Sorbs studies. To analyse the impact of tissue on heterogeneity of random effects estimates, a joint tissue analysis and a PBMC-only analysis was conducted and respective heterogeneity estimates (measured as I^2^) were compared. Panels A-C show the distributions of the I^2^ estimates as boxplots (A) and histograms (B-C), Panels D and E compare the results of the most frequently associating metabolite, namely acetylcarnitine (C2). While panel D shows the I^2^estimates of associations significant (hierarchical FDR=5%) in the PBMCs-only analysis (n=644) but not in the joint tissue analysis, panel E displays the statistics for associations reaching significance in the joint-tissue analysis only (n=1,506). As expected, loss of significance in joint analysis can be attributed to tissue heterogeneity.

## Supplemental Figure S8


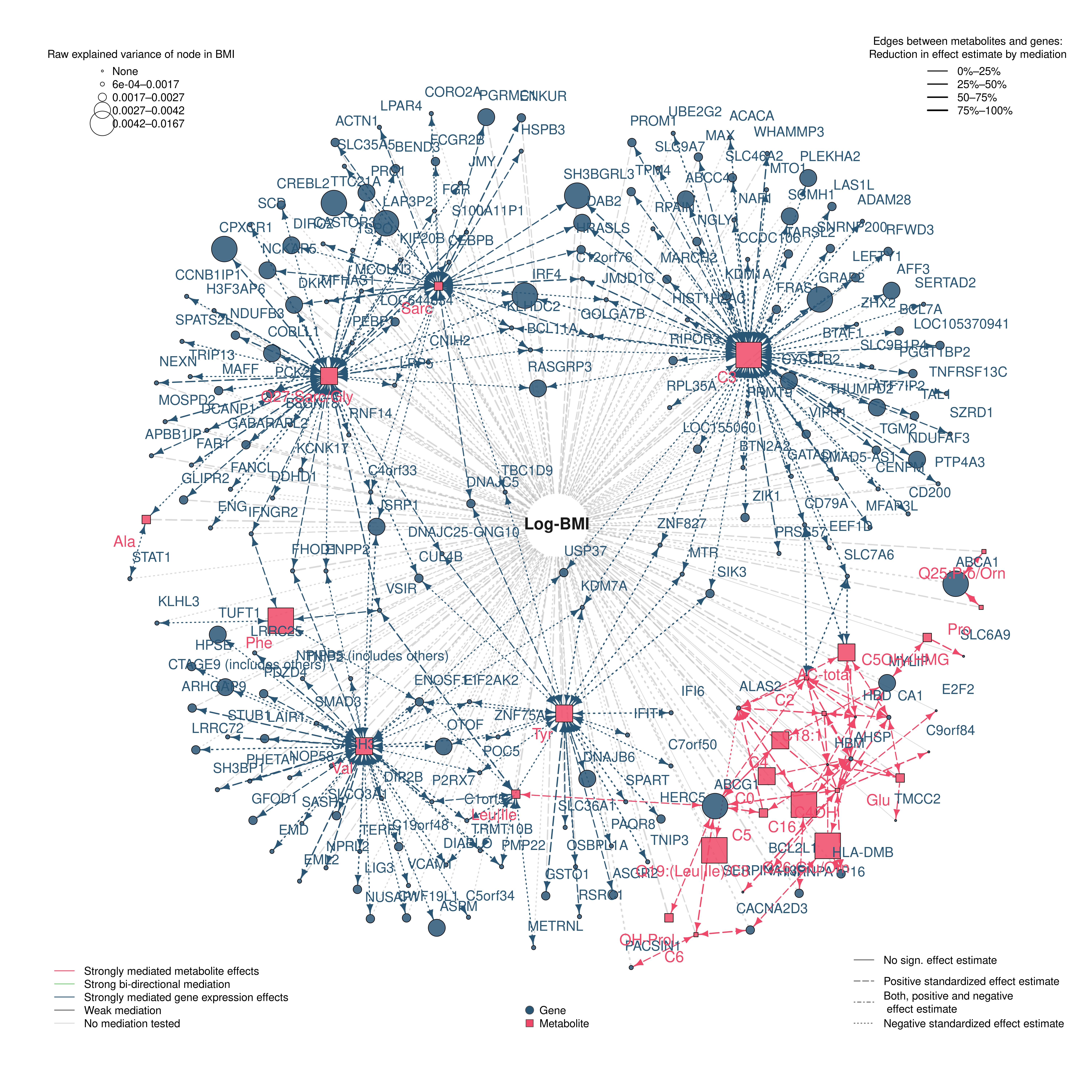


Supplemental Figure 8 Network of mediation effects on BMI. Vertices represent individual metabolites and genes, edges represent significant mediations between connected vertices. Arrows represent the direction of the mediation, with the origin as the exposure and the arrow pointing at the mediator. Grey lines from a vertex to the center represent significant associations. Coloring indicates mediations with >20% of the total effect being mediated. Due to size restrictions, the 250 strongest mediations (sorted by absolute mediation effect size) per exposure/mediator group (see color-coding) are shown.

## Supplemental Figure S9


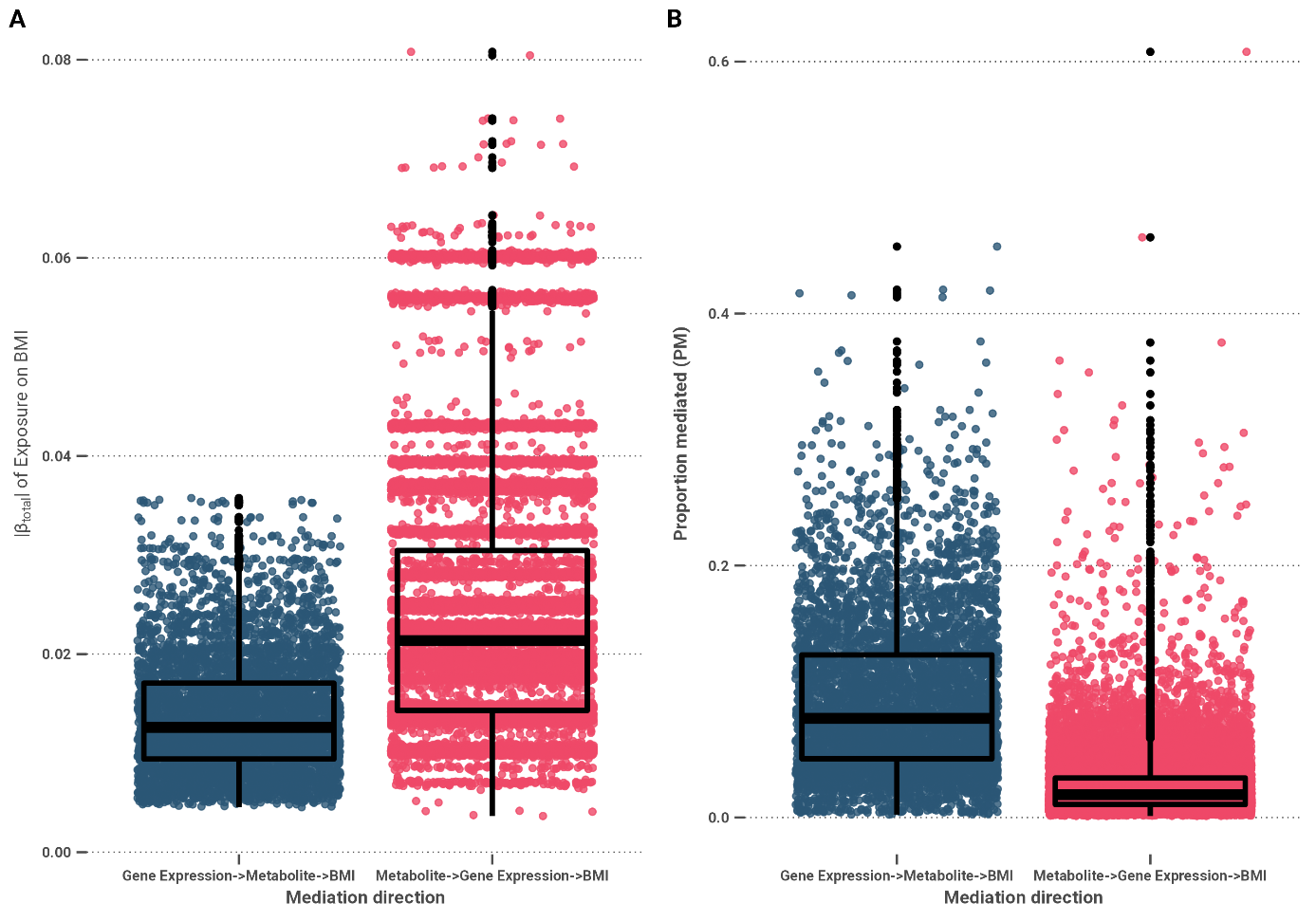


Supplemental Figure 9 Distributions of the total effect (calculated as the sum of the direct and indirect effect) of the exposure on the outcome BMI (panel A) and the mediated proportion of this effect (PM, panel B) from a mediation analysis of effects of metabolites and gene-expression on BMI. In each panel, the mediation effect and PM estimates are shown for each tested mediation direction, gene expression🡪metabolite🡪BMI (GE🡪M🡪BMI) and metabolite🡪gene expression🡪BMI (M🡪GE🡪BMI). While the overall total effects of the GE🡪M🡪BMI analysis are smaller than in the M🡪GE🡪BMI analysis, the PM of these effects are larger. Thus, the effects of gene expression on BMI are generally smaller than the effects of metabolites on BMI, but are mediated to a larger extent (by metabolites), while the larger metabolite effects are mediated to a smaller extent by gene expression effects.

References

Holdt, Lesca M.; Beutner, Frank; Scholz, Markus; Gielen, Stephan; Gäbel, Gábor; Bergert, Hendrik et al. (2010): ANRIL expression is associated with atherosclerosis risk at chromosome 9p21. In: *Arteriosclerosis, thrombosis, and vascular biology* 30 (3), S. 620–627. DOI: 10.1161/ATVBAHA.109.196832.

Holdt, Lesca M.; Hoffmann, Steve; Sass, Kristina; Langenberger, David; Scholz, Markus; Krohn, Knut et al. (2013): Alu elements in ANRIL non-coding RNA at chromosome 9p21 modulate atherogenic cell functions through trans-regulation of gene networks. In: *PLoS genetics* 9 (7), e1003588. DOI: 10.1371/journal.pgen.1003588.

Johnson, W. Evan; Li, Cheng; Rabinovic, Ariel (2007): Adjusting batch effects in microarray expression data using empirical Bayes methods. In: *Biostatistics (Oxford, England)* 8 (1), S. 118–127. DOI: 10.1093/biostatistics/kxj037.

Leek, Jeffrey T.; Johnson, W. Evan; Parker, Hilary S.; Jaffe, Andrew E.; Storey, John D. (2012): The sva package for removing batch effects and other unwanted variation in high-throughput experiments. In: *Bioinformatics* 28 (6), S. 882–883. DOI: 10.1093/bioinformatics/bts034.

Schmid, Ramona; Baum, Patrick; Ittrich, Carina; Fundel-Clemens, Katrin; Huber, Wolfgang; Brors, Benedikt et al. (2010): Comparison of normalization methods for Illumina BeadChip HumanHT-12 v3. In: *BMC genomics* 11, S. 349. DOI: 10.1186/1471-2164-11-349.
