# Supplementary material for: An atlas of genome-wide gene expression and metabolite associations and possible mediation effects towards body mass index
Source: J Mol Med (Berl). 2023 Sep 6;101(10):1305–21. doi: 10.1007/s00109-023-02362-z (PMC10560167; doi:10.1007/s00109-023-02362-z)
Supplement: Supplementary file 3 — Supplementary file1 (DOCX 11.3 KB) [file 109_2023_2362_MOESM3_ESM.docx]

https://doi.org/10.5281/zenodo.7104774
